# Discrete Sliding Mode Control Design for Bilateral Teleoperation System via Adaptive Extended State Observer

**DOI:** 10.3390/s20185091

**Published:** 2020-09-07

**Authors:** Yongli Yan, Li Ding, Yana Yang, Fucai Liu

**Affiliations:** 1 Beijing Advanced Innovation Center for Biomedical Engineering, Beihang University, Beijing 100191, China; yanyongli0318@buaa.edu.cn; 2 School of Biological Science and Medical Engineering, Beihang University, Beijing 100191, China; 3 The Institute of Electrical Engineering, Yanshan University, Qinhuangdao 066004, China; yyn@ysu.edu.cn (Y.Y.); lfc@ysu.edu.cn (F.L.)

**Keywords:** teleoperation system, discrete sliding mode, adaptive extended state observer, task space

## Abstract

The goal of this paper is to improve the synchronization control performance of nonlinear teleoperation systems with system uncertainties in the presence of time delays. In view of the nonlinear discrete states of the teleoperation system in packet-switched communication networks, a new discrete sliding mode control (DSMC) strategy is performed via a new reaching law in task space. The new reaching law is designed to reduce the chattering and improve control performance. Moreover, an adaptive extended state observer (AESO) is used to estimate the total system disturbances. The additional gain of AESO is adjusted in time to decrease the estimation errors of both system states and disturbances automatically and improve the estimation performances of the AESO. Finally, the validity of the designed control strategy is demonstrated by both simulation and experiments. Furthermore, the experimental comparison results indicate that the improvement is achievable with the proposed AESO and DSMC.

## 1. Introduction

Nonlinear bilateral teleoperation systems can expand the range of perception, and enable humans to complete complex tasks in a remote operating environment. A representative nonlinear bilateral teleoperation system consists of the following five parts: human operator, master robot, the network communication channel, slave robot, and remote environment. In recent years, the potential applications of teleoperation systems are in the various fields, i.e., the remote handling of hazardous materials [1], underwater maintenance and repairing tasks [2], space exploration [3], telemedicine [4], and so on. In all of these applications, the tasks occur in long-distance and/or hazardous environments. However, due to the limitation of bandwidth for the communication, there will be inevitable time delays. As we all know, time delay is one of the factors influencing the stability of nonlinear bilateral teleoperation systems. In practice, because of the extremely complicated nonlinearity of the nonlinear bilateral teleoperation systems and the certain constraint conditions on their outputs or states, nonlinear bilateral teleoperation systems havedifficultly in performing ideal performances. 

Nowadays, a series of effective control strategies have been performed to solve the stability problems of nonlinear bilateral teleoperation systems. In [5], a notion of wave variable had been developed to handle the time delay issue. In [6], the instability, caused by time delays, had been conquered with a new passivity and scattering theory. In [7], a robust adaptive control algorithm is developed to deal with the system’s uncertainties and to provide a smooth estimation of delayed reference signals. In [8], Baranitha tackled the time-varying delay problem for a single-master multi-slave teleoperation system by assuming an asymmetric and semi-Markovian jump protocol for communication of the slaves with the master. There were also the passivity-based assumptions employed to ensure the stability of the position and velocity of the teleoperation systems, such as [9, 10]. After that, Lu proposes a relative impedance-based force control method for a bimanual robot teleoperation system with varying time delays in [11]. Lu adopts a strategies that design a hybrid error item to minimize both the position and force tracking errors. Additionally, in order to reduce the tracking error and ensure the stability of the system, an asymmetrical wave variable compensation method is proposed [12], where the forward wave variable compensates the backward wave variable. However, the abovementioned research methods only work for joint space-based teleoperation systems. When the slave and the master are kinematically different; for instance, the slave robot is bigger/smaller than another one. Previous controllers in the joint space cannot bring about a satisfactory working performance. In order to make the robot end effector reach the desired position in the task space, Takegaki and Arimoto proposed a new position control method-based Jacobian task space transition matrix [13]. With the development of this study, many methodologies have been widely described in the literature to aim at task space-based teleoperation system control such as the new nonlinear saturated proportional derivative (SPD) control strategy based on approximate Jacobian matrix [14], a novel (nP+D)-like controller for task-space tracking performance [15], nonlinear adaptive saturation control strategy with limited amplitude [16], adaptive control schemes based on assumed parametric linearization of kinematics and dynamics [17], and so on. It is worth noting that there is no full discussion about the system uncertainties and external disturbances in the above literature, although the system stability and synchronization performances were achieved. Indeed, the above-mentioned consequences are performed under certain assumptions where the models for the communication network and the master/slave controllers have been supposed to be in the continuous-time domain. In reality, the communication media are composed of undependable communication networks that may have variable delays, lost or reordered packets. Therefore, there is an urgent need for nonlinear bilateral teleoperation in developing a discrete-time theory. 

It is well known that sliding mode control (SMC) has effective control performance for both linear and nonlinear systems, and provides more noticeable robustness and simpler calculation than other robust control approaches [18]. However, the computation of the computer is based on discrete sample systems. If the continuous-time SMC algorithms are applied directly to discrete-time systems, it is of concern that there would be some indomitable problems such as chattering and discretization errors. Therefore, the design of discrete sliding mode control (DSMC) has attracted a lot of attention in recent years. There are also some contributions in the area of DSMC such as [19]. Better performance could be guaranteed by considering a sampling period in the design phase, even if the sampling period was quite long [20]. In [21], Ma developed a novel dead zone sliding mode reaching law with disturbance compensation for uncertain discrete-time systems. A new adaptive second order DSMC scheme is proposed for a class of uncertain nonlinear systems [22]; however, better robustness and trace performance come at the cost of slightly more complex control logic. In [23], an optimal sliding surface coupled with a delay predictor had been presented to construct a DSMC for overcoming system parametric uncertainty. Moreover, we concentrate particularly on the active disturbance rejection control (ADRC). In the ADRC strategy, the extended state observer (ESO) is adopted to obtain the real-time estimation of the lumped system uncertainties including both internal unmodeled dynamics and external disturbance in the system [24]. In [25], a third-order super-twisting extended state observer was proposed, which enhanced the estimation convergence and provided strong disturbance estimation against fast speed and load variation. Liu [26] proposed that an improved model predictive control (MPC) combined with extended state observe exhibits better control performance and faster dynamic response, where the ESO provides real-time disturbance compensation for the prediction control algorithm. Consequently, in this paper, we propose a reasonable method to effectively handle total system disturbances is by introducing ESOs for the uncertain teleoperation system.

Since the communication between the master and the slave is achieved through a packet-switched network, this paper focuses specifically on the synchronization control for discrete-time nonlinear bilateral teleoperation system in task space, which includes internal unmodeled dynamics, external disturbance and time-delays. Therefore, this paper is aimed at developing a new discrete sliding mode control algorithm to ensure the synchronization performance between the master and the slave via applying the adaptive extended state observer (AESO) to compensate total disturbances. The main accomplishments of the proposed strategy are summarized as follows: (i) with the aim of avoiding discretization after control design, a discrete sliding mode control algorithm is designed, in order to consider the discrete states caused by unreliable communication networks; (ii) a new reaching law of DSMC is developed to reduce the chattering while ensuring the tracking error quickly converges to zero domain; (iii) The parameter θ of AESO is designed so that the estimation errors quickly converge to smaller neighborhood and experimental comparisons demonstrate better. Finally, the proposed control method is simulated and tested by an experiment executed on a nonlinear bilateral teleoperation system composed of two Phantom Premium 1.5A robot manipulators. The test results reveal that the proposed control algorithm yields remarkable control performance. 

The rest of this paper is organized as following. The concerned background is discussed in Section 2. In Section 3, we propose AESO-based DSMC and present the stability analyses of the nonlinear bilateral teleoperation system based on the designed controllers. Section 4 shows the simulation and experiment results. The concluding remarks are given in Section 5.

## 2. Problem Statement and Preliminaries

In this section, a $n^{1}$-degree-of-freedom (DOF) master-slave nonlinear bilateral telerobotic system is considered as follows:

(1) $$\left\{ \begin{array}{l} M_{q_{m}}(q_{m}){\ddot{q}}_{m}+C_{q_{m}}(q_{m},{\dot{q}}_{m}){\dot{q}}_{m}+g_{q_{m}}(q_{m})+f_{q_{m}}({\dot{q}}_{m})+B_{q_{m}}(q_{m})=\tau_{m}+J_{m}^{T}\left(q_{m}\right)F_{h}\\ M_{q_{s}}(q_{s}){\ddot{q}}_{s}+C_{q_{s}}(q_{s},{\dot{q}}_{s}){\dot{q}}_{s}+g_{q_{s}}(q_{s})+f_{q_{s}}({\dot{q}}_{s})+B_{q_{s}}(q_{s})=\tau_{s}-J_{s}^{T}\left(q_{s}\right)F_{e} \end{array}\right.$$

where m and s denote the master side and the slave side of the nonlinear bilateral teleoperation system, respectively, $q_{i}\in R^{n^{1}}$ with i=m,s is the joint position vector, ${\dot{q}}_{i}\in R^{n^{1}}$ is the joint velocity vector, ${\ddot{q}}_{i}\in R^{n^{1}}$ is the joint accelerated velocity vector, $M_{q_{i}}\left(q_{i}\right)\in R^{n^{1}\times n^{1}}$ is the positive-definite inertia matrix, $C_{q_{i}}\left(q_{i},{\dot{q}}_{i}\right)\in R^{n^{1}\times n^{1}}$ is the matrix of centripetal and Coriolis term, $g_{q_{i}}\left(q_{i}\right)\in R^{n^{1}}$ is the gravitational vector, $f_{q_{i}}\left({\dot{q}}_{i}\right)\in R^{n^{1}}$ is the viscous friction vector, $B_{q_{i}}\left(q_{i}\right)\in R^{n^{1}}$ denotes the unknown bounded external disturbance, $\tau_{i}\in R^{n^{1}}$ is control torque and $\tau_{i}=J_{i}^{J}\left(q_{i}\right)u_{i}$, $u_{i}\in R^{n}$ is applied input control vector, $J_{i}\left(q_{i}\right)\in R^{n\times n^{1}}$ is Jacobian matrix, and $F_{h},F_{e}\in R^{n}$ denote master operator force and external environmental force, respectively.

We review the properties [27,28,29] for teleoperation system as follows:

**Property** **1.** The inertia matrix M(q) is a symmetric positive-definite matrix, and there are two positive constants $m_{1}$ and $m_{2}$ such that $m_{1}\leq M\left(q\right)\leq m_{2}$.

**Property** **2.** There exists a positive scalar b such that ‖C(q,x)y‖≤b‖x‖‖y‖, with regard to all $q,x,y\in R^{n}$.

**Property** **3.** If $\ddot{q}$ and $\dot{q}$ are bounded, the time derivative of the term $C\left(q,\dot{q}\right)$ is also bounded.

**Assumption** **1.** *In this article, the Jacobian matrix $J_{i}\left(q_{i}\right),i=m,s$ is supposed to be known and that the robot arms are working in a finite task space; in other words, the Jacobian matrix has full rank.*

Let $\chi_{m},\chi_{s}\in R^{n}$ represent the task coordinates of the task-space end effectors. The positional relationship between task space and joint space can be represented by the following relation

(2) $$\chi_{i}=h_{i}\left(q_{i}\right),{\dot{\chi }}_{i}=J_{i}\left(q_{i}\right){\dot{q}}_{i}$$

where i=m,s, $h_{i}\left(\cdot \right):R^{n}\to R^{n}$ represents the mapping relationship from joint space to task space, and $J_{i}^{}=\partial h_{i}\left(q_{i}\right)/\partial q_{i}$.

Then, ${\dot{q}}_{i},{\dot{q}}_{i}$ are expressed as follows:

(3) $$\begin{array}{l} {\dot{q}}_{i}=J_{i}^{-1}\left(q_{i}\right){\dot{\chi }}_{i}\\ q_{i}={\dot{J}}_{i}^{-1}\left(q_{i}\right){\dot{\chi }}_{i}+J_{i}^{-1}\left(q_{i}\right){\ddot{\chi }}_{i} \end{array}$$

After the model transformation, in the task space, the system (1) can be rewritten as:

(4) $$\begin{array}{l} M_{m}{\ddot{\chi }}_{m}+C_{m}{\dot{\chi }}_{m}+g_{m}+f_{m}+B_{m}=u_{m}+F_{h}\\ M_{s}{\ddot{\chi }}_{s}+C_{s}{\dot{\chi }}_{s}+g_{s}+f_{s}+B_{s}=u_{s}-F_{e} \end{array}$$

where i=m,s,

$$\begin{array}{l} M_{i}={\left(J_{i}^{T}\left(q_{i}\right)\right)}^{-1}M_{q_{i}}\left(q_{i}\right)J_{i}^{-1}\left(q_{i}\right)\\ C_{i}={\left(J_{i}^{T}\left(q_{i}\right)\right)}^{-1}\left(M_{q_{i}}\left(q_{i}\right){\dot{J}}_{i}^{-1}\left(q_{i}\right)+C_{q_{i}}\left(q_{i},{\dot{q}}_{i}\right)J_{i}^{-1}\left(q_{i}\right)\right)\\ g_{i}={\left(J_{i}^{T}\left(q_{i}\right)\right)}^{-1}g_{q_{i}}\left(q_{i}\right)\\ f_{i}={\left(J_{i}^{T}\left(q_{i}\right)\right)}^{-1}f_{q_{i}}\left(q_{i}\right)\\ B_{i}={\left(J_{i}^{T}\left(q_{i}\right)\right)}^{-1}B_{q_{i}}\left(q_{i}\right) \end{array}$$

In most practical applications, the precise mode cannot be obtained directly, due to the noise, friction, viscous friction, uncertain disturbances and so on. Due to the existence of certain uncertainties in $M_{i}$ and $C_{i}$, the system (4) is rewritten as follows:

(5) $$\begin{array}{l} M_{\mathrm{om}}{\ddot{\chi }}_{m}+C_{\mathrm{om}}{\dot{\chi }}_{m}-M_{\mathrm{om}}\Theta_{m}=u_{m}\\ M_{\mathrm{os}}{\ddot{\chi }}_{s}+C_{\mathrm{os}}{\dot{\chi }}_{s}-M_{\mathrm{os}}\Theta_{s}=u_{s} \end{array}$$

where $M_{i}=M_{oi}+\Delta M_{i}$, $C_{i}=C_{oi}+\Delta C_{i}$, $\Theta_{i}=-M_{oi}^{-1}\left(\Delta M_{i}{\ddot{\chi }}_{i}+\Delta C_{i}{\dot{\chi }}_{i}+g_{i}+f_{i}+B_{i}-F_{i}\right)$, i=m,s. $M_{oi}$, $C_{oi}$ represent the nominal parts, while $\Delta M_{i}$ and $\Delta C_{i}$ represent the uncertainties. When i=m, $F_{i}=F_{h}$, otherwise, $F_{i}=-F_{e}$. In this paper, $\Theta_{i}$ denotes the lumped system uncertainty, and it is assumed to be bounded. 

Introducing the state vector $X_{i}={\left[\begin{array}{cc} X_{i1}^{T} & X_{i2}^{T} \end{array}\right]}^{T}$, i=m,s, let $X_{m1}=\chi_{m}$, $X_{m2}={\dot{\chi }}_{m}$, $X_{s1}=\chi_{s}$, $X_{s2}={\dot{\chi }}_{s}$, then the system (5) is transformed as follows:

(6) $$\begin{array}{l} {\dot{X}}_{i1}=X_{i2}\\ {\dot{X}}_{i2}=f_{i}\left(X_{i}\right)+\Theta_{i}+H_{i}u_{i} \end{array}$$

Furthermore, the system (6) is rewritten as follows:

(7) $${\dot{X}}_{i}=AX_{i}+Bf_{i}\left(X_{i}\right)+B\Theta_{i}+BH_{i}u_{i}$$

where i=m,s, $A=\left[\begin{array}{cc} 0 & I\\ 0 & 0 \end{array}\right]$, $B=\left[\begin{array}{c} 0\\ I \end{array}\right]$, $H_{i}=M_{oi}^{-1}$.

Notice that all the terms proposed in (6) and (7) can be easily calculated from (5). With the sampling time h, the discretization of the uncertain model equation is given as:

(8) $$X_{i}\left(jh+h\right)={\bar{A}}_{i}X_{i}\left(jh\right)+{\bar{B}}_{i}f_{i}\left(jh\right)+{\bar{B}}_{i}\Theta_{i}\left(jh\right)+{\bar{B}}_{i}H_{i}u_{i}\left(jh\right)$$

where

$${\bar{A}}_{i}=\exp\left(\left[A_{i}\right]h\right)=\left[\begin{array}{cc} I & hI\\ 0 & I \end{array}\right]$$

$${\bar{B}}_{i}=\int_{0}^{h}\exp\left(\left[A_{i}\right]h\right)d\gamma \left[B_{i}\right]=\left[\begin{array}{c} \frac{h^{2}}{2!}I\\ hI \end{array}\right]$$

## 3. Main Results

This section addresses a presentation of a proposed control scheme based on discrete-time nonlinear bilateral teleoperation system, where the new AESO is introduced to estimate and compensate the uncertainty. Meanwhile, the development of a discrete-time SMC algorithm is depicted, with a view to settle the synchronization problem of bilateral teleoperation system in task space.

### 3.1. Adaptive Extended State Observer

In this part, the adaptive extended state observer will be employed for the system (9). Firstly, for the convenience of further analysis, the following state measurement values are introduced:

(9) $$Y_{i}\left(jh\right)=X_{i1}\left(jh\right)+n_{ij}$$

where i=m,s $X_{i1}\left(jh\right)$ is the output to be controlled, and $Y_{i}\left(jh\right)\in R^{n}$ is the one to be measured, which includes the measurement noise vector $n_{ij}\in R^{n}$. Then, the following assumptions for the observer are given as [30]:

**Assumption** **2.** ${\left\{n_{ij}\right\}}_{1}^{\infty }$*is a white random sequence and*

(10) $$E\left(n_{ij}n_{ij}^{T}\right)\leq {\bar{R}}_{i}$$

*where ${\bar{R}}_{i}$ is a known matrix.*

**Assumption** **3.** (11) $$E\left[\begin{array}{c} X_{i}\left(0\right)-{\hat{X}}_{i}\left(0\right)\\ \Theta_{i}\left(0\right)-{\hat{\Theta }}_{i}\left(0\right) \end{array}\right]{\left[\begin{array}{c} X_{i}\left(0\right)-{\hat{X}}_{i}\left(0\right)\\ \Theta_{i}\left(0\right)-{\hat{\Theta }}_{i}\left(0\right) \end{array}\right]}^{T}\leq P_{i0}$$

*where $\left[\begin{array}{c} X_{i}\left(0\right)-{\hat{X}}_{i}\left(0\right)\\ \Theta_{i}\left(0\right)-{\hat{\Theta }}_{i}\left(0\right) \end{array}\right]$ is the estimation error of the AESO, and $P_{i0}$ is a known matrix.*

**Assumption** **4.** (12) $$E\left(\Theta_{i}\left(X_{i}\left(t\right),t\right)-\Theta_{i}\left(X_{i}\left(jh\right),jh\right)\right){\left(\Theta_{i}\left(X_{i}\left(t\right),t\right)-\Theta_{i}\left(X_{i}\left(jh\right),jh\right)\right)}^{T}\leq {\bar{Q}}_{i}$$

*where t∈[jh,jh+h), j≥0, $\Theta_{i}\left(X_{i}\left(t\right),t\right)-\Theta_{i}\left(X_{i}\left(jh\right),jh\right)$ is the discretized error and ${\bar{Q}}_{i}$ is a known diagonal matrix.*

Therefore, the linear structure of AESO is designed as follows:

(13) $$\left[\begin{array}{c} {\hat{X}}_{i}\left(jh+h\right)\\ {\hat{\Theta }}_{i}\left(jh+h\right) \end{array}\right]={\tilde{A}}_{i}\left[\begin{array}{c} {\hat{X}}_{i}\left(jh\right)\\ {\hat{\Theta }}_{i}\left(jh\right) \end{array}\right]+{\tilde{B}}_{i}H_{i}u_{i}\left(jh\right)+{\tilde{B}}_{i}f_{i}\left(jh\right)+L_{ij}\left(Y_{i}\left(jh\right)-{\hat{X}}_{i1}\left(jh\right)\right)$$

where ${\tilde{A}}_{i}=\exp\left(\left[\begin{array}{cc} A_{i} & B_{i}\\ 0 & 0 \end{array}\right]h\right)=\left[\begin{array}{ccc} I & hI & \frac{h^{2}}{2!}I\\ 0 & I & hI\\ 0 & 0 & I \end{array}\right]$, ${\tilde{B}}_{i}=\int_{0}^{h}\exp\left(\left[\begin{array}{cc} A_{i} & B_{i}\\ 0 & 0 \end{array}\right]h\right)d\gamma \left[\begin{array}{c} B_{i}\\ 0 \end{array}\right]=\left[\begin{array}{c} \frac{h^{2}}{2!}I\\ hI\\ 0 \end{array}\right]$, and $L_{ij}$ is the gain of the discrete AESO, ensuring that ${\hat{X}}_{i}\left(jh\right)$ and ${\hat{\Theta }}_{i}\left(jh\right)$ can be employed as the estimation of $X_{i}\left(jh\right)$ and $\Theta_{i}\left(jh\right)$, respectively. Then, the original values of AESO (13) are taken as:

(14) $${\hat{X}}_{i1}\left(0\right)=Y_{i}\left(0\right),{\hat{X}}_{ik}\left(0\right)=0,k\geq 2,{\hat{\Theta }}_{i}\left(0\right)=0$$

Therefore, the gain $L_{ij}$ of the AESO is designed as follows:

(15) $$L_{ij}={\tilde{A}}_{i}\left(I+\theta_{i}\right)P_{ij}C_{i}{\left(C_{i}^{T}\left(I+\theta_{i}\right)P_{ij}C_{i}+{\bar{R}}_{i}\right)}^{-1}$$

(16) $$P_{i\left(j+1\right)}=\left({\tilde{A}}_{i}-L_{ij}C_{i}^{T}\right)\left(I+\theta_{i}\right)P_{ij}{\left({\tilde{A}}_{i}-L_{ij}C_{i}^{T}\right)}^{T}+L_{ij}{\bar{R}}_{i}L_{ij}^{T}+\left(I+\theta_{i}^{-1}\right){\bar{Q}}_{i}$$

where

(17) $$Q_{i}=3n{\left[\begin{array}{ccc} h^{4}{\bar{Q}}_{i} & 0 & 0\\ 0 & h^{2}{\bar{Q}}_{i} & 0\\ 0 & 0 & {\bar{Q}}_{i} \end{array}\right]}_{3n\times 3n},\;C_{i}={\left[\begin{array}{c} I\\ 0\\ 0 \end{array}\right]}_{3\left(n+1\right)\times n}$$

And $eig\left(\theta_{i}\right){\left[\begin{array}{cccc} \theta_{i1} & \theta_{i2} & \cdots & \theta_{i\left(3n\right)} \end{array}\right]}^{T}$, $\theta_{i\bar{k}}=\sqrt{Q_{i}\left(\bar{k},\bar{k}\right)/P_{i0}\left(\bar{k},\bar{k}\right)}$, $\bar{k}=1,2,\ldots ,3n$

Define the estimation error $\xi_{i}\left(jh\right)$ as follows:

(18) $$\xi_{i}\left(jh\right)=\left[\begin{array}{c} X_{i}\left(jh\right)-{\hat{X}}_{i}\left(jh\right)\\ \Theta_{i}\left(X_{i}\left(jh\right),jh\right)-{\hat{\Theta }}_{i}\left(jh\right) \end{array}\right]$$

then, we will provide the following Theorem 1 showing the property of the estimation error $\xi_{i}\left(jh\right)$.

**Theorem** **1.** *If Assumptions 2–4 hold, and there exist the positive real numbers a, c, $p_{1}$, $p_{2}$, $\varepsilon_{1}$, $\varepsilon_{2}$, $\bar{\theta }$, q and r as following:*

(19) $$\begin{array}{ll} \left\|{\tilde{A}}_{i}\right\|=a & \left\|\theta_{i}\right\|=\bar{\theta }\\ \left\|C_{i}\right\|=c & \left\|Q_{i}\right\|=q\\ p_{1}I\leq P_{ij}\leq p_{2}I & \left\|{\bar{R}}_{i}\right\|=r\\ \varepsilon_{1}\leq \left\|\xi_{i}\left(jh\right)\right\|\leq \varepsilon_{2} & \end{array}$$

*so that the inequality is fulfilled:*

(20) $$ο=\frac{1}{p_{1}}\left(a+\left(1+\bar{\theta }\right)ap_{2}c^{2}/r\right)\left(q+p_{2}\right)+\frac{q}{p_{1}}+\frac{{\left(1+\bar{\theta }\right)}^{2}a^{2}p_{2}^{2}c^{2}}{p_{1}r}<\frac{a\varepsilon_{1}^{2}}{p_{2}}$$

*Then the estimation error ${\left\{\xi_{i}\left(jh\right)\right\}}_{j=0}^{\infty }$ will be uniformly bounded in the mean square, if the initial estimation error $\xi_{i}\left(0\right)$ satisfies:*

(21) $$\left\|\xi_{i}\left(0\right)\right\|\leq \varepsilon_{2}$$

Before proof of **Theorem 1**, the following lemma is first discussed.

**Lemma** **1** **([31]).** *In the case of **Theorem 1**, there is a real number 0<α<1 such that $\Pi_{ij}=P_{ij}^{-1}$ meet the inequality:*

(22) $${\left({\tilde{A}}_{i}-L_{ij}C_{i}^{T}\right)}^{T}\Pi_{i\left(j+1\right)}\left({\tilde{A}}_{i}-L_{ij}C_{i}^{T}\right)\leq \left(1-\alpha \right)\Pi_{ij}$$

*For j≥0 with $L_{ij}$ given by (15).*

**Proof.**  From (16), we have:

(23) $$\begin{array}{ll} P_{i\left(j+1\right)} & =\left({\tilde{A}}_{i}-L_{ij}C_{i}^{T}\right)\left(I+\theta_{i}\right)P_{ij}{\left({\tilde{A}}_{i}-L_{ij}C_{i}^{T}\right)}^{T}+L_{ij}{\bar{R}}_{i}L_{ij}^{T}+\left(I+\theta_{i}^{-1}\right){\bar{Q}}_{i}\\ & =\left({\tilde{A}}_{i}-L_{ij}C_{i}^{T}\right)[\left(I+\theta_{i}\right)P_{ij}+{\left({\tilde{A}}_{i}-L_{ij}C_{i}^{T}\right)}^{-1}(L_{ij}{\bar{R}}_{i}L_{ij}^{T}\\ & +\left(I+\theta_{i}^{-1}\right){\bar{Q}}_{i}){\left({\tilde{A}}_{i}-L_{ij}C_{i}^{T}\right)}^{-T}]{\left({\tilde{A}}_{i}-L_{ij}C_{i}^{T}\right)}^{T} \end{array}$$

From (19), and $C_{i}^{T}\left(I+\theta_{i}\right)P_{ij}C_{i}>0$, we have:

(24) $$\left\|L_{ij}\right\|\leq \left(1+\bar{\theta }\right)ap_{2}c\frac{1}{r}$$

since, we get:

(25) $$P_{i\left(j+1\right)}\geq \left({\tilde{A}}_{i}-L_{ij}C_{i}^{T}\right)\left[\left(I+\theta_{i}\right)P_{ij}+\frac{{\left(1+\bar{\theta }\right)}^{2}a^{2}p_{2}^{2}c^{2}/r+\left(1+{\bar{\theta }}^{-1}\right)q}{{\left(a+\left(1+\bar{\theta }\right)ap_{2}c^{2}/r\right)}^{2}}I\right]{\left({\tilde{A}}_{i}-L_{ij}C_{i}^{T}\right)}^{T}$$

Because of $P_{ij}\geq p_{1}I$, invert both sides of this inequality (25), multiply both sides by ${\left({\tilde{A}}_{i}-L_{ij}C_{i}^{T}\right)}^{T}$ and $\left({\tilde{A}}_{i}-L_{ij}C_{i}^{T}\right)$, then we finally obtain:

(26) $${\left({\tilde{A}}_{i}-L_{ij}C_{i}^{T}\right)}^{T}\Pi_{i\left(j+1\right)}\left({\tilde{A}}_{i}-L_{ij}C_{i}^{T}\right)\leq {\left[1+\bar{\theta }+\frac{{\left(1+\bar{\theta }\right)}^{2}a^{2}p_{2}^{2}c^{2}/r+\left(1+{\bar{\theta }}^{-1}\right)q}{{\left(a+\left(1+\bar{\theta }\right)ap_{2}c^{2}/r\right)}^{2}}\right]}^{-1}\Pi_{ij}$$

Therefore:

(27) $$1-\alpha ={\left[1+\bar{\theta }+\frac{{\left(1+\bar{\theta }\right)}^{2}a^{2}p_{2}^{2}c^{2}/r+\left(1+{\bar{\theta }}^{-1}\right)q}{{\left(a+\left(1+\bar{\theta }\right)ap_{2}c^{2}/r\right)}^{2}}\right]}^{-1}$$

 □

**Proof of Theorem** **1.** From (7) and (13), we have:

(28) $$\xi_{i}\left(jh+h\right)=\left({\tilde{A}}_{i}-L_{ij}C_{i}^{T}\right)\xi_{i}\left(jh\right)+L_{ij}n_{ij}+\left[\begin{array}{c} W_{ij}\\ G_{ij} \end{array}\right]$$

where:

$$W_{ij}=\int_{jh}^{\left(j+1\right)h}\left[\begin{array}{c} \frac{{\left(\left(j+1\right)h-\gamma \right)}^{n-1}}{n-1}I\\ \vdots \\ \frac{\left(\left(j+1\right)h-\gamma \right)}{1}I\\ I \end{array}\right]\left(\Theta_{i}\left(X_{i}\left(t\right),t\right)-\Theta_{i}\left(X_{i}\left(jh\right),jh\right)\right)d\gamma$$

$$G_{ij}=\Theta_{i}\left(jh+h\right)-\Theta_{i}\left(jh\right)$$

and the last term of Equation (28) satisfies:

(29) $$E\left[\begin{array}{c} W_{ij}\\ G_{ij} \end{array}\right]{\left[\begin{array}{c} W_{ij}\\ G_{ij} \end{array}\right]}^{T}\leq Q_{i}$$

Define $\Gamma_{ij}=E\left(\xi_{i}\left(jh\right)\xi_{i}{\left(jh\right)}^{T}\right)$, thus:

(30) $$\begin{array}{ll} \Gamma_{i\left(j+1\right)} & =\left({\tilde{A}}_{i}-L_{ij}C_{i}^{T}\right)\Gamma_{ij}{\left({\tilde{A}}_{i}-L_{ij}C_{i}^{T}\right)}^{T}\\ & +E\left[\begin{array}{c} W_{ij}\\ G_{ij} \end{array}\right]{\left[\begin{array}{c} W_{ij}\\ G_{ij} \end{array}\right]}^{T}+\left({\tilde{A}}_{i}-L_{ij}C_{i}^{T}\right)E\xi_{i}\left(jh\right){\left(L_{ij}n_{ij}+\left[\begin{array}{c} W_{ij}\\ G_{ij} \end{array}\right]\right)}^{T}\\ & +E\left(L_{ij}n_{ij}+\left[\begin{array}{c} W_{ij}\\ G_{ij} \end{array}\right]\right)\xi_{i}^{T}\left(jh\right){\left({\tilde{A}}_{i}-L_{ij}C_{i}^{T}\right)}^{T}+L_{ij}En_{ij}n_{ij}^{T}L_{ij}^{T} \end{array}$$

With the above knowable, the measurement noise vector $n_{ij}$ and the estimation error $\xi_{i}\left(jh\right)$ are unrelated, so the following inequality is given as:

(31) $$\begin{array}{ll} \Gamma_{i\left(j+1\right)} & \leq \left({\tilde{A}}_{i}-L_{ij}C_{i}^{T}\right)\Gamma_{ij}{\left({\tilde{A}}_{i}-L_{ij}C_{i}^{T}\right)}^{T}\\ & +E\left[\begin{array}{c} W_{ij}\\ G_{ij} \end{array}\right]{\left[\begin{array}{c} W_{ij}\\ G_{ij} \end{array}\right]}^{T}+\left({\tilde{A}}_{i}-L_{ij}C_{i}^{T}\right)E{\left(\xi_{i}\left(jh\right)\left[\begin{array}{c} W_{ij}\\ G_{ij} \end{array}\right]\right)}^{T}\\ & +E\left(\left[\begin{array}{c} W_{ij}\\ G_{ij} \end{array}\right]\xi_{i}^{T}\left(jh\right)\right){\left({\tilde{A}}_{i}-L_{ij}C_{i}^{T}\right)}^{T}+L_{ij}{\bar{R}}_{i}L_{ij}^{T} \end{array}$$

According to **Assumption 4**, the last third and second terms of (31) have the following upper bound:

(32) $$\begin{array}{c} \left({\tilde{A}}_{i}-L_{ij}C_{i}^{T}\right)E{\left(\xi_{i}\left(jh\right)\left[\begin{array}{c} W_{ij}\\ G_{ij} \end{array}\right]\right)}^{T}+E\left(\left[\begin{array}{c} W_{ij}\\ G_{ij} \end{array}\right]\xi_{i}^{T}\left(jh\right)\right){\left({\tilde{A}}_{i}-L_{ij}C_{i}^{T}\right)}^{T}\\ \leq \left({\tilde{A}}_{i}-L_{ij}C_{i}^{T}\right)\theta_{i}\Gamma_{ij}{\left({\tilde{A}}_{i}-L_{ij}C_{i}^{T}\right)}^{T}+\theta_{i}^{-1}Q_{i} \end{array}$$

Note that the proper $\theta_{i}$ can make the two sides of the inequality as close as possible. If and only if j=1, the equation of (32) can be achieved. In consequence, we get the following equation:

(33) $$\left({\tilde{A}}_{i}-L_{i0}C_{i}^{T}\right)\theta_{i}E\xi_{i}\left(0\right)\xi_{i}^{T}\left(0\right){\left({\tilde{A}}_{i}-L_{i0}C_{i}^{T}\right)}^{T}=\theta_{i}^{-1}E\left[\begin{array}{c} W_{i0}\\ G_{i0} \end{array}\right]{\left[\begin{array}{c} W_{i0}\\ G_{i0} \end{array}\right]}^{T}$$

where:

(34) $$E\xi_{i}\left(0\right)\xi_{i}^{T}\left(0\right)=P_{i0},\;E\left[\begin{array}{c} W_{i0}\\ G_{i0} \end{array}\right]{\left[\begin{array}{c} W_{i0}\\ G_{i0} \end{array}\right]}^{T}=Q_{i}$$

Because of ${\tilde{A}}_{i}-L_{i0}C_{i}^{T}\approx I$, the Equation (33) and Equation (34) indicate that:

(35) $$\theta_{i}^{2}=Q_{i}P_{i0}^{-1}$$

Since $Q_{i}$ and $P_{i0}^{}$ are diagonal matrices, we set:

(36) $$eig\left(\theta_{i}\right){\left[\begin{array}{cccc} \theta_{i1} & \theta_{i2} & \cdots & \theta_{i\left(3n\right)} \end{array}\right]}^{T},\;\theta_{i\bar{k}}=\sqrt{Q_{i}\left(\bar{k},\bar{k}\right)/P_{i0}\left(\bar{k},\bar{k}\right)},\;\bar{k}=1,2,\ldots ,3n$$

 □

**Remark** **1.** *For the parameter $\theta_{i}^{}$, if it is chosen as a constant, each item of gain $L_{ij}$ is iterated in the same way. Therefore, it is possible that the corresponding gain for the derivative of total disturbance estimation is large, which could easily lead to an overgrowth of the total disturbance estimation. Consequently, the gain of controller would be set sufficiently large to stabilize the closed-loop teleoperation system. Unfortunately, this will come with the actuator saturation problem. In order to solve this problem, the new parameter $\theta_{i}^{}$ in the form of a diagonal matrix, is proposed in this paper. In other words, parameters are adjusted, respectively, according to the variation of different system states in this paper. Therefore, each state could reach its ideal one as quickly as possible, then the stability will be guaranteed.*

Next:

(37) $$\Gamma_{i\left(j+1\right)}\leq \left({\tilde{A}}_{i}-L_{ij}C_{i}^{T}\right)\left(I+\theta_{i}^{}\right)\Gamma_{ij}{\left({\tilde{A}}_{i}-L_{ij}C_{i}^{T}\right)}^{T}+L_{ij}{\bar{R}}_{i}L_{ij}^{T}+\left(I+\theta_{i}^{-1}\right)Q_{i}$$

Thus:

(38) $$P_{i\left(j+1\right)}=\left({\tilde{A}}_{i}-L_{ij}C_{i}^{T}\right)\left(I+\theta_{i}^{}\right)\Gamma_{ij}{\left({\tilde{A}}_{i}-L_{ij}C_{i}^{T}\right)}^{T}+L_{ij}{\bar{R}}_{i}L_{ij}^{T}+\left(I+\theta_{i}^{-1}\right)Q_{i}$$

and $P_{i0}\geq \Gamma_{i0}$, we get:

(39) $$\Gamma_{i\left(j+1\right)}\leq P_{i\left(j+1\right)},\;j\geq 0$$

Therefore, $P_{i\left(j+1\right)}$ is regarded as the upper bound of the covariance matrix of $\xi_{i}\left(jh+h\right)$, and it is also recommended to be minimized by proposing $L_{ij}$. Design

(40) $$J_{ij}=trace\left(P_{i\left(j+1\right)}\right)$$

When the partial derivative of $J_{ij}$ with respect to $L_{ij}$ is zero, that is:

(41) $$\frac{\partial J_{ij}}{\partial L_{ij}}=0$$

It is easy to know

(42) $$L_{ij}={\tilde{A}}_{i}\left(I+\theta_{i}\right)P_{ij}C_{i}{\left(C_{i}^{T}\left(I+\theta_{i}\right)P_{ij}C_{i}+{\bar{R}}_{i}\right)}^{-1}$$

We design Lyapunov function as follows:

(43) $$V_{ij}=\xi_{i}^{T}\left(jh\right)\Pi_{ij}\xi_{i}\left(jh\right)$$

From (28), we have:

(44) $$\begin{array}{l} V_{i\left(j+1\right)}=\\ {\left[\left({\tilde{A}}_{i}-L_{ij}C_{i}^{T}\right)\xi_{i}\left(jh\right)+L_{ij}n_{ij}+\left[\begin{array}{c} W_{ij}\\ G_{ij} \end{array}\right]\right]}^{T}\Pi_{i\left(j+1\right)}\left[\left({\tilde{A}}_{i}-L_{ij}C_{i}^{T}\right)\xi_{i}\left(jh\right)+L_{ij}n_{ij}+\left[\begin{array}{c} W_{ij}\\ G_{ij} \end{array}\right]\right] \end{array}$$

and using Lemma 1 and Equation (43), the Equation (44) can be transformed as:

(45) $$\begin{array}{ll} V_{i\left(j+1\right)} & \leq \left(1-\alpha \right)V_{ij}\\ & +{\left[\begin{array}{c} W_{ij}\\ G_{ij} \end{array}\right]}^{T}\Pi_{i\left(j+1\right)}\left[\left({\tilde{A}}_{i}-L_{ij}C_{i}^{T}\right)\xi_{i}\left(jh\right)+L_{ij}n_{ij}\right]\\ & +\xi_{i}^{T}\left(jh\right){\left({\tilde{A}}_{i}-L_{ij}C_{i}^{T}\right)}^{T}\Pi_{i\left(j+1\right)}\left(L_{ij}n_{ij}+\left[\begin{array}{c} W_{ij}\\ G_{ij} \end{array}\right]\right)\\ & +n_{ij}^{T}L_{ij}^{T}\Pi_{i\left(j+1\right)}\left(\left({\tilde{A}}_{i}-L_{ij}C_{i}^{T}\right)\xi_{i}\left(jh\right)+\left[\begin{array}{c} W_{ij}\\ G_{ij} \end{array}\right]\right)\\ & +{\left[\begin{array}{c} W_{ij}\\ G_{ij} \end{array}\right]}^{T}\Pi_{i\left(j+1\right)}\left[\begin{array}{c} W_{ij}\\ G_{ij} \end{array}\right]+n_{ij}^{T}L_{ij}^{T}\Pi_{i\left(j+1\right)}L_{ij}n_{ij} \end{array}$$

Since $\xi_{i}\left(jh\right)$ is not related to $n_{ij}$, we can get further:

(46) $$\begin{array}{ll} V_{i\left(j+1\right)} & \leq \left(1-\alpha \right)V_{ij}+{\left[\begin{array}{c} W_{ij}\\ G_{ij} \end{array}\right]}^{T}\Pi_{i\left(j+1\right)}\left({\tilde{A}}_{i}-L_{ij}C_{i}^{T}\right)\xi_{i}\left(jh\right)\\ & +\xi_{i}^{T}\left(jh\right){\left({\tilde{A}}_{i}-L_{ij}C_{i}^{T}\right)}^{T}\Pi_{i\left(j+1\right)}\left[\begin{array}{c} W_{ij}\\ G_{ij} \end{array}\right]\\ & +{\left[\begin{array}{c} W_{ij}\\ G_{ij} \end{array}\right]}^{T}\Pi_{i\left(j+1\right)}\left[\begin{array}{c} W_{ij}\\ G_{ij} \end{array}\right]+n_{ij}^{T}L_{ij}^{T}\Pi_{i\left(j+1\right)}L_{ij}n_{ij} \end{array}$$

For the second and third terms of the (46), apply the (19) and we will have:

(47) $$\begin{array}{l} {\left[\begin{array}{c} W_{ij}\\ G_{ij} \end{array}\right]}^{T}\Pi_{i\left(j+1\right)}\left({\tilde{A}}_{i}-L_{ij}C_{i}^{T}\right)\xi_{i}\left(jh\right)+\xi_{i}^{T}\left(jh\right){\left({\tilde{A}}_{i}-L_{ij}C_{i}^{T}\right)}^{T}\Pi_{i\left(j+1\right)}\left[\begin{array}{c} W_{ij}\\ G_{ij} \end{array}\right]\\ \;\leq \frac{1}{p_{1}}\left(a+\left(1+\bar{\theta }\right)ap_{2}c^{2}/r\right)\left({\left[\begin{array}{c} W_{ij}\\ G_{ij} \end{array}\right]}^{T}\xi_{i}\left(jh\right)+\xi_{i}^{T}\left(jh\right)\left[\begin{array}{c} W_{ij}\\ G_{ij} \end{array}\right]\right)\\ \;\leq \frac{1}{p_{1}}\left(a+\left(1+\bar{\theta }\right)ap_{2}c^{2}/r\right)\left({\left[\begin{array}{c} W_{ij}\\ G_{ij} \end{array}\right]}^{T}\left[\begin{array}{c} W_{ij}\\ G_{ij} \end{array}\right]+\xi_{i}^{T}\left(jh\right)\xi_{i}\left(jh\right)\right)\\ \;\leq \frac{1}{p_{1}}\left(a+\left(1+\bar{\theta }\right)ap_{2}c^{2}/r\right)\left(q+p_{2}\right) \end{array}$$

For the fourth term of the (46), apply the (19) and we will have:

(48) $$\begin{array}{ll} {\left[\begin{array}{c} W_{ij}\\ G_{ij} \end{array}\right]}^{T}\Pi_{i\left(j+1\right)}\left[\begin{array}{c} W_{ij}\\ G_{ij} \end{array}\right] & \leq \frac{1}{p_{1}}{\left[\begin{array}{c} W_{ij}\\ G_{ij} \end{array}\right]}^{T}\left[\begin{array}{c} W_{ij}\\ G_{ij} \end{array}\right]\\ & \leq \frac{q}{p_{1}} \end{array}$$

and for the fifth term of the (46), apply the (19) and we will have

(49) $$n_{ij}^{T}L_{ij}^{T}\Pi_{i\left(j+1\right)}L_{ij}n_{ij}\leq \frac{1}{p_{1}}n_{ij}^{T}L_{ij}^{T}L_{ij}n_{ij}$$

Since both sides of (49) are scalars, we can trace the right side of (49) and at the same time will not change its value, that is

(50) $$\begin{array}{ll} n_{ij}^{T}L_{ij}^{T}\Pi_{i\left(j+1\right)}L_{ij}n_{ij} & \leq \frac{1}{p_{1}}tr\left(n_{ij}^{T}L_{ij}^{T}L_{ij}n_{ij}\right)\\ & \leq \frac{1}{p_{1}}tr\left(L_{ij}n_{ij}n_{ij}^{T}L_{ij}^{T}\right)\\ & \leq {\frac{{\left(1+\bar{\theta }\right)}^{2}a^{2}p_{2}^{2}c}{p_{1}r}}^{2} \end{array}$$

Finally, synthesize the inequalities (46)–(50) above and we can get:

(51) $$V_{i\left(j+1\right)}-V_{ij}\leq -\alpha V_{ij}+ο$$

where $ο=\frac{1}{p_{1}}\left(a+\left(1+\bar{\theta }\right)ap_{2}c^{2}/r\right)\left(q+p_{2}\right)+\frac{q}{p_{1}}+{\frac{{\left(1+\bar{\theta }\right)}^{2}a^{2}p_{2}^{2}c}{p_{1}r}}^{2}$. When $ο<\frac{a\varepsilon_{1}^{2}}{p_{2}}$, using (19) and (43), we obtain:

(52) $$V_{i\left(j+1\right)}-V_{ij}\leq 0$$

From discussion above, we may conclude that the appropriate positive parameters a, c, $p_{1}$, $p_{2}$, $\varepsilon_{1}$, $\varepsilon_{2}$, $\bar{\theta }$, q and r may be utilized to guarantee the stability of the error system (143), and the estimation error $\xi_{i}\left(jh\right)$ converges to a smaller zero domain.

### 3.2. Discrete Sliding Mode Surface

In the above subsection, the lumped system uncertainty of the bilateral teleoperation system is estimated by the new AESO (13). In this part, its estimation is employed as the compensation for the uncertainty, and DSMC is provided by the following main theorem. For the nonlinear bilateral teleoperation system, tracking trajectories of master and slave robots are $X_{\mathrm{dm}}\left(jh\right)=X_{s}\left(jh-T_{s}\right)$ and $X_{\mathrm{ds}}\left(jh\right)=X_{m}\left(jh-T_{m}\right)$, respectively, where $T_{i},i=m,s$ is the constant time delay. $T_{m}$ represents the time delay of signal transmission from the master robot to the slave robot and $T_{s}$ stands for the time delay of signal transmission from the slave robot to the master robot. So, the position synchronization errors between the master and the slave are defined as follows:

(53) $$\begin{array}{l} e_{m}\left(jh\right)=X_{m}\left(jh\right)-X_{\mathrm{dm}}\left(jh\right)\\ e_{s}\left(jh\right)=X_{s}\left(jh\right)-X_{\mathrm{ds}}\left(jh\right) \end{array}$$

Next, a switching surface is constructed, and the dynamic equation of the ideal sliding mode is obtained by using the equivalent control method. Then, sufficient conditions for the asymptotic dynamic stability of the sliding mode are obtained by using Lyapunov stability theory and LMI (Linear Matrix Inequality) method.

Select the following switching function:

(54) $$s_{i}\left(jh\right)=\Lambda_{i}e_{i}\left(jh\right)$$

with $\Lambda_{i}\in R^{n\times 2n}$, i=m,s, which is assumed that $\Lambda_{i}{\bar{B}}_{i}\neq 0$. Then

(55) $$\begin{array}{ll} s_{i}\left(jh+h\right) & =\Lambda_{i}e_{i}\left(jh+h\right)\\ & =\Lambda_{i}{\bar{A}}_{i}X_{i}\left(jh\right)+\Lambda_{i}{\bar{B}}_{i}f_{i}\left(jh\right)+\Lambda_{i}{\bar{B}}_{i}\Theta_{i}\left(jh\right)\\ & \;+\Lambda_{i}{\bar{B}}_{i}H_{i}u_{i}\left(jh\right)-\Lambda_{i}X_{di}\left(jh+h\right) \end{array}$$

According to the discrete sliding mode theory, the equation $s_{i}\left(jh+h\right)=s_{i}\left(jh\right)=0$ is obtained when the system states reach the switching surface. Therefore, the ideal sliding mode equivalent control law can be obtained from formulas (8) and (55) as follows:

(56) $$\begin{array}{ll} u_{i}^{\mathrm{eq}}\left(jh\right)= & -{\left(\Lambda_{i}{\bar{B}}_{i}H_{i}\right)}^{-1}(\Lambda_{i}{\bar{A}}_{i}X_{i}\left(jh\right)+\Lambda_{i}{\bar{B}}_{i}f_{i}\left(jh\right)\\ & +\Lambda_{i}{\bar{B}}_{i}\Theta_{i}\left(jh\right)-\Lambda_{i}X_{di}\left(jh+h\right)) \end{array}$$

**Note** **1.** *The equivalent control law shown in Equation (57) is only used to analyze the stability of the sliding mode dynamic equation, and it is only used as a tool here. The actual sliding mode control law will be designed below.*

By substituting the equivalent control law (56) into the system (8), the sliding mode dynamic equation of the uncertain discrete time-delay system under ideal conditions can be obtained as follows:

(57) $$\begin{array}{ll} X_{i}\left(jh+h\right) & =\left({\bar{A}}_{i}-{\bar{B}}_{i}H_{i}{\left(\Lambda_{i}{\bar{B}}_{i}H_{i}\right)}^{-1}\Lambda_{i}{\bar{A}}_{i}\right)X_{i}\left(jh\right)\\ & +\left({\bar{B}}_{i}-{\bar{B}}_{i}H_{i}{\left(\Lambda_{i}{\bar{B}}_{i}H_{i}\right)}^{-1}\Lambda_{i}{\bar{B}}_{i}\right)f_{i}\left(jh\right)\\ & +\left({\bar{B}}_{i}-{\bar{B}}_{i}H_{i}{\left(\Lambda_{i}{\bar{B}}_{i}H_{i}\right)}^{-1}\Lambda_{i}{\bar{B}}_{i}\right)\Theta_{i}\left(jh\right)\\ & +{\bar{B}}_{i}H_{i}{\left(\Lambda_{i}{\bar{B}}_{i}H_{i}\right)}^{-1}\Lambda_{i}X_{di}\left(jh+h\right) \end{array}$$

The stability of the sliding mode dynamic equation (58) is further analyzed by using the LMI technique, and sufficient conditions are given to guarantee the asymptotic stability of the sliding mode dynamic equation.

**Theorem** **2.** *For uncertain discrete time-delay system (8), switch function (54) is selected. If there exist positive definite matrix $P_{i}>0$ and scalar $\varpi_{i1}$, $\varpi_{i2}$, and the following LMI inequalities (58)-(60) are satisfied, then the sliding mode dynamic system (57) is asymptotically stable:*

(58) $$\left[\begin{array}{ccc} \Xi_{i} & 2{ϒ}_{i}^{T}P_{i} & \sqrt{8}{\left({ϒ}_{i}{\bar{A}}_{i}\right)}^{T}P_{i}\\ 2P_{i}{ϒ}_{i}^{} & -P_{i} & 0\\ \sqrt{8}P_{i}{ϒ}_{i}{\bar{A}}_{i} & 0 & -P_{i} \end{array}\right]<0$$

(59) $$\left[\begin{array}{cc} -P & P{\bar{B}}_{i}E\\ E^{T}{\bar{B}}_{i}^{T}P & -\varpi_{i1}I \end{array}\right]<0$$

(60) $$\left[\begin{array}{cc} -P_{i} & P_{i}{ϒ}_{i}{\bar{B}}_{i}E\\ E^{T}{\left({ϒ}_{i}{\bar{B}}_{i}^{}\right)}^{T}P_{i} & -\varpi_{i2}I \end{array}\right]<0$$

*where i=m,s, $\Xi_{i}=8{\bar{A}}_{i}^{T}P_{i}{\bar{A}}_{i}-P_{i}+8\varpi_{i1}\left(D_{f_{i}}^{T}D_{f_{i}}^{}+D_{\Theta_{i}}^{T}D_{\Theta_{i}}^{}\right)+8\varpi_{i2}\left(D_{f_{i}}^{T}D_{f_{i}}^{}+D_{\Theta_{i}}^{T}D_{\Theta_{i}}^{}\right)$, ${ϒ}_{i}={\bar{B}}_{i}H_{i}{\left(\Lambda_{i}{\bar{B}}_{i}H_{i}\right)}^{-1}\Lambda_{i}$.*

**Assumption** **5.** $f_{i}$ *and $\Theta_{i}$ represent the nonlinear part of the teleoperation system and the disturbance, so the following condition is satisfied:*

(61) $$\left[\begin{array}{cc} f_{i}\left(jh\right) & \Theta_{i}\left(jh\right) \end{array}\right]=EF\left(jh\right)\left[\begin{array}{cc} D_{f_{i}} & D_{\Theta_{i}} \end{array}\right]$$

*where E, $D_{f_{i}}$, $D_{\Theta_{i}}$ and F(jh) are known constant matrices, and F(jh) satisfies $F^{T}\left(jh\right)F\left(jh\right)\leq I$.*

**Lemma** **2** **([32]).** *If E and D are the real matrixes with the appropriate dimensions, and F(jh) satisfies $F^{T}\left(jh\right)F\left(jh\right)\leq I$. Then, for any non-zero constant ϖ>0, the following inequality exists:*

(62) $$EF\left(jh\right)D+D^{T}F^{T}\left(jh\right)E^{T}=\varpi^{-1}E^{T}E+\varpi D^{T}D$$

**Lemma** **3** **([32]).** *If ν and υ are the real matrixes with the appropriate dimensions, for any non-zero matrix U>0, then*

(63) $$\nu \upsilon +\nu^{T}\upsilon^{T}=\nu U\nu^{T}+\upsilon^{T}U^{-1}\upsilon$$

**Lemma** **4** **([33]).**  *(Schur’s theorem) For the following LMI inequality*

(64) $$\left[\begin{array}{cc} \Omega_{11} & \Omega_{12}\\ \Omega_{12} & -\Omega_{22} \end{array}\right]<0$$

*where $\Omega_{22}>0$, $\Omega_{11}+\Omega_{12}\Omega_{22}^{-1}\Omega_{12}<0$, so this is the same thing as $\Omega_{11}=\Omega_{11}^{T}$, $\Omega_{22}=\Omega_{22}^{T}$.*

**Proof.**  In this proof, to facilitate the proof process, the hypothesizes and lemmas above are given. □

Let us take the Lyapunov function as

(65) $$V_{i}\left(jh\right)=X_{i}^{T}\left(jh\right)P_{i}X_{i}\left(jh\right)$$

Along the state trajectory of the system (57), it can be obtained that:

(66) $$\begin{array}{ll} \Delta V_{i}\left(jh\right) & =V_{i}\left(jh+h\right)-V_{i}\left(jh\right)\\ & =\Delta V_{i1}\left(jh\right)+\Delta V_{i2}\left(jh\right) \end{array}$$

where

$$\begin{array}{ll} \Delta V_{i1}\left(jh\right) & =X_{i}^{T}\left(jh\right){\left({\bar{A}}_{i}-{\bar{B}}_{i}H_{i}{\left(\Lambda_{i}{\bar{B}}_{i}H_{i}\right)}^{-1}\Lambda_{i}{\bar{A}}_{i}\right)}^{T}P_{i}\left({\bar{A}}_{i}-{\bar{B}}_{i}H_{i}{\left(\Lambda_{i}{\bar{B}}_{i}H_{i}\right)}^{-1}\Lambda_{i}{\bar{A}}_{i}\right)X_{i}\left(jh\right)\\ & +f_{i}^{T}\left(jh\right){\left({\bar{B}}_{i}-{\bar{B}}_{i}H_{i}{\left(\Lambda_{i}{\bar{B}}_{i}H_{i}\right)}^{-1}\Lambda_{i}{\bar{B}}_{i}\right)}^{T}P_{i}\left({\bar{B}}_{i}-{\bar{B}}_{i}H_{i}{\left(\Lambda_{i}{\bar{B}}_{i}H_{i}\right)}^{-1}\Lambda_{i}{\bar{B}}_{i}\right)f_{i}\left(jh\right)\\ & +\Theta_{i}^{T}\left(jh\right){\left({\bar{B}}_{i}-{\bar{B}}_{i}H_{i}{\left(\Lambda_{i}{\bar{B}}_{i}H_{i}\right)}^{-1}\Lambda_{i}{\bar{B}}_{i}\right)}^{T}P_{i}\left({\bar{B}}_{i}-{\bar{B}}_{i}H_{i}{\left(\Lambda_{i}{\bar{B}}_{i}H_{i}\right)}^{-1}\Lambda_{i}{\bar{B}}_{i}\right)\Theta_{i}\left(jh\right)\\ & +X_{di}^{T}\left(jh+h\right){\left({\bar{B}}_{i}H_{i}{\left(\Lambda_{i}{\bar{B}}_{i}H_{i}\right)}^{-1}\Lambda_{i}\right)}^{T}P_{i}\left({\bar{B}}_{i}H_{i}{\left(\Lambda_{i}{\bar{B}}_{i}H_{i}\right)}^{-1}\Lambda_{i}\right)X_{di}\left(jh+h\right)\\ & -X_{i}^{T}\left(jh\right)P_{i}X_{i}\left(jh\right) \end{array}$$

$$\begin{array}{ll} \Delta V_{i2}\left(jh\right) & =X_{i}^{T}\left(jh\right){\left({\bar{A}}_{i}-{\bar{B}}_{i}H_{i}{\left(\Lambda_{i}{\bar{B}}_{i}H_{i}\right)}^{-1}\Lambda_{i}{\bar{A}}_{i}\right)}^{T}P_{i}\left({\bar{B}}_{i}-{\bar{B}}_{i}H_{i}{\left(\Lambda_{i}{\bar{B}}_{i}H_{i}\right)}^{-1}\Lambda_{i}{\bar{B}}_{i}\right)f_{i}\left(jh\right)\\ & +f_{i}^{T}\left(jh\right){\left({\bar{B}}_{i}-{\bar{B}}_{i}H_{i}{\left(\Lambda_{i}{\bar{B}}_{i}H_{i}\right)}^{-1}\Lambda_{i}{\bar{B}}_{i}\right)}^{T}P_{i}\left({\bar{A}}_{i}-{\bar{B}}_{i}H_{i}{\left(\Lambda_{i}{\bar{B}}_{i}H_{i}\right)}^{-1}\Lambda_{i}{\bar{A}}_{i}\right)X_{i}\left(jh\right)\\ & +X_{i}^{T}\left(jh\right){\left({\bar{A}}_{i}-{\bar{B}}_{i}H_{i}{\left(\Lambda_{i}{\bar{B}}_{i}H_{i}\right)}^{-1}\Lambda_{i}{\bar{A}}_{i}\right)}^{T}P_{i}\left({\bar{B}}_{i}-{\bar{B}}_{i}H_{i}{\left(\Lambda_{i}{\bar{B}}_{i}H_{i}\right)}^{-1}\Lambda_{i}{\bar{B}}_{i}\right)\Theta_{i}\left(jh\right)\\ & +\Theta_{i}^{T}\left(jh\right){\left({\bar{B}}_{i}-{\bar{B}}_{i}H_{i}{\left(\Lambda_{i}{\bar{B}}_{i}H_{i}\right)}^{-1}\Lambda_{i}{\bar{B}}_{i}\right)}^{T}P_{i}\left({\bar{A}}_{i}-{\bar{B}}_{i}H_{i}{\left(\Lambda_{i}{\bar{B}}_{i}H_{i}\right)}^{-1}\Lambda_{i}{\bar{A}}_{i}\right)X_{i}\left(jh\right)\\ & +X_{i}^{T}\left(jh\right){\left({\bar{A}}_{i}-{\bar{B}}_{i}H_{i}{\left(\Lambda_{i}{\bar{B}}_{i}H_{i}\right)}^{-1}\Lambda_{i}{\bar{A}}_{i}\right)}^{T}P_{i}\left({\bar{B}}_{i}H_{i}{\left(\Lambda_{i}{\bar{B}}_{i}H_{i}\right)}^{-1}\Lambda_{i}\right)X_{di}\left(jh\right)\\ & +X_{di}^{T}\left(jh\right){\left({\bar{B}}_{i}H_{i}{\left(\Lambda_{i}{\bar{B}}_{i}H_{i}\right)}^{-1}\Lambda_{i}\right)}^{T}P_{i}\left({\bar{A}}_{i}-{\bar{B}}_{i}H_{i}{\left(\Lambda_{i}{\bar{B}}_{i}H_{i}\right)}^{-1}\Lambda_{i}{\bar{A}}_{i}\right)X_{i}\left(jh\right)\\ & +f_{i}^{T}\left(jh\right){\left({\bar{B}}_{i}-{\bar{B}}_{i}H_{i}{\left(\Lambda_{i}{\bar{B}}_{i}H_{i}\right)}^{-1}\Lambda_{i}{\bar{B}}_{i}\right)}^{T}P_{i}\left({\bar{B}}_{i}-{\bar{B}}_{i}H_{i}{\left(\Lambda_{i}{\bar{B}}_{i}H_{i}\right)}^{-1}\Lambda_{i}{\bar{B}}_{i}\right)\Theta_{i}\left(jh\right)\\ & +\Theta_{i}^{T}\left(jh\right){\left({\bar{B}}_{i}-{\bar{B}}_{i}H_{i}{\left(\Lambda_{i}{\bar{B}}_{i}H_{i}\right)}^{-1}\Lambda_{i}{\bar{B}}_{i}\right)}^{T}P_{i}\left({\bar{B}}_{i}-{\bar{B}}_{i}H_{i}{\left(\Lambda_{i}{\bar{B}}_{i}H_{i}\right)}^{-1}\Lambda_{i}{\bar{B}}_{i}\right)f_{i}\left(jh\right)\\ & +f_{i}^{T}\left(jh\right){\left({\bar{B}}_{i}-{\bar{B}}_{i}H_{i}{\left(\Lambda_{i}{\bar{B}}_{i}H_{i}\right)}^{-1}\Lambda_{i}{\bar{B}}_{i}\right)}^{T}P_{i}\left({\bar{B}}_{i}H_{i}{\left(\Lambda_{i}{\bar{B}}_{i}H_{i}\right)}^{-1}\Lambda_{i}\right)X_{di}\left(jh\right)\\ & +X_{di}^{T}\left(jh\right){\left({\bar{B}}_{i}H_{i}{\left(\Lambda_{i}{\bar{B}}_{i}H_{i}\right)}^{-1}\Lambda_{i}\right)}^{T}P_{i}\left({\bar{B}}_{i}-{\bar{B}}_{i}H_{i}{\left(\Lambda_{i}{\bar{B}}_{i}H_{i}\right)}^{-1}\Lambda_{i}{\bar{B}}_{i}\right)f_{i}\left(jh\right)\\ & +\Theta_{i}^{T}\left(jh\right){\left({\bar{B}}_{i}-{\bar{B}}_{i}H_{i}{\left(\Lambda_{i}{\bar{B}}_{i}H_{i}\right)}^{-1}\Lambda_{i}{\bar{B}}_{i}\right)}^{T}P_{i}\left({\bar{B}}_{i}H_{i}{\left(\Lambda_{i}{\bar{B}}_{i}H_{i}\right)}^{-1}\Lambda_{i}\right)X_{di}\left(jh\right)\\ & +X_{di}^{T}\left(jh\right){\left({\bar{B}}_{i}-{\bar{B}}_{i}H_{i}{\left(\Lambda_{i}{\bar{B}}_{i}H_{i}\right)}^{-1}\Lambda_{i}{\bar{B}}_{i}\right)}^{T}P_{i}\left({\bar{B}}_{i}H_{i}{\left(\Lambda_{i}{\bar{B}}_{i}H_{i}\right)}^{-1}\Lambda_{i}\right)\Theta_{i}\left(jh\right) \end{array}$$

For the first and second terms of the expression $\Delta V_{i2}\left(jh\right)$, it can be seen from the **Lemma 3** that:

(67) $$\begin{array}{c} X_{i}^{T}\left(jh\right){\left({\bar{A}}_{i}-{\bar{B}}_{i}H_{i}{\left(\Lambda_{i}{\bar{B}}_{i}H_{i}\right)}^{-1}\Lambda_{i}{\bar{A}}_{i}\right)}^{T}P_{i}\left({\bar{B}}_{i}-{\bar{B}}_{i}H_{i}{\left(\Lambda_{i}{\bar{B}}_{i}H_{i}\right)}^{-1}\Lambda_{i}{\bar{B}}_{i}\right)f_{i}\left(jh\right)\\ +f_{i}^{T}\left(jh\right){\left({\bar{B}}_{i}-{\bar{B}}_{i}H_{i}{\left(\Lambda_{i}{\bar{B}}_{i}H_{i}\right)}^{-1}\Lambda_{i}{\bar{B}}_{i}\right)}^{T}P_{i}\left({\bar{A}}_{i}-{\bar{B}}_{i}H_{i}{\left(\Lambda_{i}{\bar{B}}_{i}H_{i}\right)}^{-1}\Lambda_{i}{\bar{A}}_{i}\right)X_{i}\left(jh\right)\\ \leq X_{i}^{T}\left(jh\right){\left({\bar{A}}_{i}-{\bar{B}}_{i}H_{i}{\left(\Lambda_{i}{\bar{B}}_{i}H_{i}\right)}^{-1}\Lambda_{i}{\bar{A}}_{i}\right)}^{T}P\left({\bar{A}}_{i}-{\bar{B}}_{i}H_{i}{\left(\Lambda_{i}{\bar{B}}_{i}H_{i}\right)}^{-1}\Lambda_{i}{\bar{A}}_{i}\right)X_{i}\left(jh\right)\\ +f_{i}^{T}\left(jh\right){\left({\bar{B}}_{i}-{\bar{B}}_{i}H_{i}{\left(\Lambda_{i}{\bar{B}}_{i}H_{i}\right)}^{-1}\Lambda_{i}{\bar{B}}_{i}\right)}^{T}P_{i}\left({\bar{B}}_{i}-{\bar{B}}_{i}H_{i}{\left(\Lambda_{i}{\bar{B}}_{i}H_{i}\right)}^{-1}\Lambda_{i}{\bar{B}}_{i}\right)f_{i}\left(jh\right) \end{array}$$

Similarly, for other terms of the expression $\Delta V_{i2}\left(jh\right)$, we can get:

(68) $$\begin{array}{l} \Delta V_{i2}\left(jh\right)\\ \;\leq 3X_{i}^{T}\left(jh\right){\left({\bar{A}}_{i}-{\bar{B}}_{i}H_{i}{\left(\Lambda_{i}{\bar{B}}_{i}H_{i}\right)}^{-1}\Lambda_{i}{\bar{A}}_{i}\right)}^{T}P_{i}\left({\bar{A}}_{i}-{\bar{B}}_{i}H_{i}{\left(\Lambda_{i}{\bar{B}}_{i}H_{i}\right)}^{-1}\Lambda_{i}{\bar{A}}_{i}\right)X_{i}\left(jh\right)\\ \;+3f_{i}^{T}\left(jh\right){\left({\bar{B}}_{i}-{\bar{B}}_{i}H_{i}{\left(\Lambda_{i}{\bar{B}}_{i}H_{i}\right)}^{-1}\Lambda_{i}{\bar{B}}_{i}\right)}^{T}P_{i}\left({\bar{B}}_{i}-{\bar{B}}_{i}H_{i}{\left(\Lambda_{i}{\bar{B}}_{i}H_{i}\right)}^{-1}\Lambda_{i}{\bar{B}}_{i}\right)f_{i}\left(jh\right)\\ \;+3\Theta_{i}^{T}\left(jh\right){\left({\bar{B}}_{i}-{\bar{B}}_{i}H_{i}{\left(\Lambda_{i}{\bar{B}}_{i}H_{i}\right)}^{-1}\Lambda_{i}{\bar{B}}_{i}\right)}^{T}P_{i}\left({\bar{B}}_{i}-{\bar{B}}_{i}H_{i}{\left(\Lambda_{i}{\bar{B}}_{i}H_{i}\right)}^{-1}\Lambda_{i}{\bar{B}}_{i}\right)\Theta_{i}\left(jh\right)\\ \;+3X_{di}^{T}\left(jh\right){\left({\bar{B}}_{i}H_{i}{\left(\Lambda_{i}{\bar{B}}_{i}H_{i}\right)}^{-1}\Lambda_{i}\right)}^{T}P_{i}\left({\bar{B}}_{i}H_{i}{\left(\Lambda_{i}{\bar{B}}_{i}H_{i}\right)}^{-1}\Lambda_{i}\right)X_{di}\left(jh\right) \end{array}$$

Therefore,

(69) $$\begin{array}{l} \Delta V_{i}\left(jh\right)\leq -X_{i}^{T}\left(jh\right)P_{i}X_{i}\left(jh\right)\\ \;+4X_{i}^{T}\left(jh\right){\left({\bar{A}}_{i}-{\bar{B}}_{i}H_{i}{\left(\Lambda_{i}{\bar{B}}_{i}H_{i}\right)}^{-1}\Lambda_{i}{\bar{A}}_{i}\right)}^{T}P_{i}\left({\bar{A}}_{i}-{\bar{B}}_{i}H_{i}{\left(\Lambda_{i}{\bar{B}}_{i}H_{i}\right)}^{-1}\Lambda_{i}{\bar{A}}_{i}\right)X_{i}\left(jh\right)\\ \;+4f_{i}^{T}\left(jh\right){\left({\bar{B}}_{i}-{\bar{B}}_{i}H_{i}{\left(\Lambda_{i}{\bar{B}}_{i}H_{i}\right)}^{-1}\Lambda_{i}{\bar{B}}_{i}\right)}^{T}P_{i}\left({\bar{B}}_{i}-{\bar{B}}_{i}H_{i}{\left(\Lambda_{i}{\bar{B}}_{i}H_{i}\right)}^{-1}\Lambda_{i}{\bar{B}}_{i}\right)f_{i}\left(jh\right)\\ \;+4\Theta_{i}^{T}\left(jh\right){\left({\bar{B}}_{i}-{\bar{B}}_{i}H_{i}{\left(\Lambda_{i}{\bar{B}}_{i}H_{i}\right)}^{-1}\Lambda_{i}{\bar{B}}_{i}\right)}^{T}P_{i}\left({\bar{B}}_{i}-{\bar{B}}_{i}H_{i}{\left(\Lambda_{i}{\bar{B}}_{i}H_{i}\right)}^{-1}\Lambda_{i}{\bar{B}}_{i}\right)\Theta_{i}\left(jh\right)\\ \;+4X_{di}^{T}\left(jh\right){\left({\bar{B}}_{i}H_{i}{\left(\Lambda_{i}{\bar{B}}_{i}H_{i}\right)}^{-1}\Lambda_{i}\right)}^{T}P_{i}\left({\bar{B}}_{i}H_{i}{\left(\Lambda_{i}{\bar{B}}_{i}H_{i}\right)}^{-1}\Lambda_{i}\right)X_{di}\left(jh\right) \end{array}$$

According to the **Lemma 3**, the following inequality is further obtained:

(70) $$\begin{array}{l} \Delta V_{i}\left(jh\right)\leq \\ X_{i}^{T}\left(jh\right)\left(8{\bar{A}}_{i}^{T}P_{i}{\bar{A}}_{i}-P_{i}+{\left({\bar{B}}_{i}H_{i}{\left(\Lambda_{i}{\bar{B}}_{i}H_{i}\right)}^{-1}\Lambda_{i}{\bar{A}}_{i}\right)}^{T}P_{i}\left({\bar{B}}_{i}H_{i}{\left(\Lambda_{i}{\bar{B}}_{i}H_{i}\right)}^{-1}\Lambda_{i}{\bar{A}}_{i}\right)\right)X_{i}\left(jh\right)\\ +f_{i}^{T}\left(jh\right)\left(8{\bar{B}}_{i}^{T}P_{i}{\bar{B}}_{i}+{\left({\bar{B}}_{i}H_{i}{\left(\Lambda_{i}{\bar{B}}_{i}H_{i}\right)}^{-1}\Lambda_{i}{\bar{B}}_{i}\right)}^{T}P_{i}\left({\bar{B}}_{i}H_{i}{\left(\Lambda_{i}{\bar{B}}_{i}H_{i}\right)}^{-1}\Lambda_{i}{\bar{B}}_{i}\right)\right)f_{i}\left(jh\right)\\ +\Theta_{i}^{T}\left(jh\right)\left(8{\bar{B}}_{i}^{T}P_{i}{\bar{B}}_{i}+{\left({\bar{B}}_{i}H_{i}{\left(\Lambda_{i}{\bar{B}}_{i}H_{i}\right)}^{-1}\Lambda_{i}{\bar{B}}_{i}\right)}^{T}P_{i}\left({\bar{B}}_{i}H_{i}{\left(\Lambda_{i}{\bar{B}}_{i}H_{i}\right)}^{-1}\Lambda_{i}{\bar{B}}_{i}\right)\right)\Theta_{i}\left(jh\right)\\ +4X_{di}^{T}\left(jh\right){\left({\bar{B}}_{i}H_{i}{\left(\Lambda_{i}{\bar{B}}_{i}H_{i}\right)}^{-1}\Lambda_{i}\right)}^{T}P_{i}\left({\bar{B}}_{i}H_{i}{\left(\Lambda_{i}{\bar{B}}_{i}H_{i}\right)}^{-1}\Lambda_{i}\right)X_{di}\left(jh\right) \end{array}$$

In addition, it can be obtained from **Assumption 5** that

(71) $$\begin{array}{l} \Delta V_{i}\left(jh\right)\leq \\ X_{i}^{T}\left(jh\right)\left(8{\bar{A}}_{i}^{T}P_{i}{\bar{A}}_{i}-P_{i}+{\left({ϒ}_{i}{\bar{A}}_{i}\right)}^{T}P_{i}\left({ϒ}_{i}{\bar{A}}_{i}\right)\right)X_{i}\left(jh\right)\\ +X_{i}^{T}\left(jh\right)D_{f_{i}}^{T}F^{T}\left(jh\right)E^{T}\left(8{\bar{B}}_{i}^{T}P_{i}{\bar{B}}_{i}+{\left({ϒ}_{i}{\bar{B}}_{i}\right)}^{T}P_{i}\left({ϒ}_{i}{\bar{B}}_{i}\right)\right)EF\left(jh\right)D_{f_{i}}^{}X_{i}\left(jh\right)\\ +X_{i}^{T}\left(jh\right)D_{\Theta_{i}}^{T}F^{T}\left(jh\right)E^{T}\left(8{\bar{B}}_{i}^{T}P_{i}{\bar{B}}_{i}+{\left({ϒ}_{i}{\bar{B}}_{i}\right)}^{T}P_{i}\left({ϒ}_{i}{\bar{B}}_{i}\right)\right)EF\left(jh\right)D_{\Theta_{i}}^{}X_{i}\left(jh\right)\\ +4X_{di}^{T}\left(jh\right){\left({ϒ}_{i}\right)}^{T}P_{i}\left({ϒ}_{i}\right)X_{di}\left(jh\right) \end{array}$$

where i=m,s, ${ϒ}_{i}={\bar{B}}_{i}H_{i}{\left(\Lambda_{i}{\bar{B}}_{i}H_{i}\right)}^{-1}\Lambda_{i}$. So, here we have

(72) $$\Delta V_{i}\left(jh\right)\leq {\left[\begin{array}{cc} X_{i}^{T}\left(jh\right) & X_{di}^{T}\left(jh\right){\left({ϒ}_{i}\right)}^{T} \end{array}\right]}^{T}\Sigma_{i}\left[\begin{array}{c} X_{i}\left(jh\right)\\ X_{di}\left(jh\right) \end{array}\right]$$

where:

$$\Sigma_{i}=\left[\begin{array}{cc} \Xi_{i1} & 0\\ 0 & \Xi_{i2} \end{array}\right]$$

$$\begin{array}{ll} \Xi_{i1} & =8{\bar{A}}_{i}^{T}P_{i}{\bar{A}}_{i}-P_{i}+{\left({ϒ}_{i}{\bar{A}}_{i}\right)}^{T}P_{i}\left({ϒ}_{i}{\bar{A}}_{i}\right)\\ & +D_{f_{i}}^{T}F^{T}\left(jh\right)E^{T}\left(8{\bar{B}}_{i}^{T}P_{i}{\bar{B}}_{i}+{\left({ϒ}_{i}{\bar{B}}_{i}\right)}^{T}P_{i}\left({ϒ}_{i}{\bar{B}}_{i}\right)\right)EF\left(jh\right)D_{f_{i}}^{}\\ & +D_{\Theta_{i}}^{T}F^{T}\left(jh\right)E^{T}\left(8{\bar{B}}_{i}^{T}P_{i}{\bar{B}}_{i}+{\left({ϒ}_{i}{\bar{B}}_{i}\right)}^{T}P_{i}\left({ϒ}_{i}{\bar{B}}_{i}\right)\right)EF\left(jh\right)D_{\Theta_{i}}^{} \end{array}$$

$$\Xi_{i2}=4{\left({ϒ}_{i}\right)}^{T}P_{i}\left({ϒ}_{i}\right)$$

Therefore, when $\Sigma_{i}<0$, $\Delta V_{i}\left(jh\right)<0$ is true (If $X_{i}\left(jh\right)\neq 0$). According to the **Lemma 4**, the inequality $\Sigma_{i}<0$ can be equivalent to:

(73) $$\begin{array}{l} {}[\begin{array}{cccc} 8{\bar{A}}_{i}^{T}P_{i}{\bar{A}}_{i}-P_{i} & 2{ϒ}_{i}^{T}P_{i} & \sqrt{8}{\left({ϒ}_{i}{\bar{A}}_{i}\right)}^{T}P_{i} & \sqrt{8}D_{f_{i}}^{T}F^{T}\left(jh\right)E^{T}{\bar{B}}_{i}^{T}P_{i}\\ 2{ϒ}_{i}P_{i} & -P_{i} & 0 & 0\\ \sqrt{8}P_{i}{ϒ}_{i}{\bar{A}}_{i} & 0 & -P_{i} & 0\\ \sqrt{8}P_{i}{\bar{B}}_{i}EF\left(jh\right)D_{f_{i}}^{} & 0 & 0 & -P_{i}\\ 0 & \sqrt{8}P_{i}{\bar{B}}_{i}EF\left(jh\right)D_{\Theta_{i}}^{} & 0 & 0\\ \sqrt{8}P_{i}{ϒ}_{i}{\bar{B}}_{i}EF\left(jh\right)D_{f_{i}}^{} & 0 & 0 & 0\\ 0 & \sqrt{8}P_{i}{ϒ}_{i}{\bar{B}}_{i}EF\left(jh\right)D_{\Theta_{i}}^{} & 0 & 0 \end{array}\\ \begin{array}{ccc} 0 & \sqrt{8}D_{f_{i}}^{T}F^{T}\left(jh\right)E^{T}{\bar{B}}_{i}^{T}{ϒ}_{i}^{T}P_{i} & 0\\ \sqrt{8}D_{\Theta_{i}}^{T}F^{T}\left(jh\right)E^{T}{\bar{B}}_{i}^{T}P_{i} & 0 & \sqrt{8}D_{\Theta_{i}}^{T}F^{T}\left(jh\right)E^{T}{\bar{B}}_{i}^{T}{ϒ}_{i}^{T}P\\ 0 & 0 & 0\\ 0 & 0 & 0\\ -P_{i} & 0 & 0\\ 0 & -P_{i} & 0\\ 0 & 0 & -P_{i} \end{array}]<0 \end{array}$$

Furthermore, the inequality (73) is equivalent to

(74) $$\begin{array}{l} {}[\begin{array}{cccc} 8{\bar{A}}_{i}^{T}P_{i}{\bar{A}}_{i}-P_{i} & 2{ϒ}_{i}^{T}P_{i} & \sqrt{8}{\left({ϒ}_{i}{\bar{A}}_{i}\right)}^{T}P_{i} & 0\\ 2{ϒ}_{i}P_{i} & -P_{i} & 0 & 0\\ \sqrt{8}P_{i}{ϒ}_{i}{\bar{A}}_{i} & 0 & -P_{i} & 0\\ 0 & 0 & 0 & -P_{i}\\ 0 & 0 & 0 & 0\\ \sqrt{8}P_{i}{ϒ}_{i}{\bar{B}}_{i}EF\left(jh\right)D_{f_{i}}^{} & 0 & 0 & 0\\ 0 & \sqrt{8}P_{i}{ϒ}_{i}{\bar{B}}_{i}EF\left(jh\right)D_{\Theta_{i}}^{} & 0 & 0 \end{array}\\ \begin{array}{ccc} 0 & \sqrt{8}D_{f_{i}}^{T}F^{T}\left(jh\right)E^{T}{\bar{B}}_{i}^{T}{ϒ}_{i}^{T}P_{i} & 0\\ 0 & 0 & \sqrt{8}D_{\Theta_{i}}^{T}F^{T}\left(jh\right)E^{T}{\bar{B}}_{i}^{T}{ϒ}_{i}^{T}P\\ 0 & 0 & 0\\ 0 & 0 & 0\\ -P_{i} & 0 & 0\\ 0 & -P_{i} & 0\\ 0 & 0 & -P_{i} \end{array}]\\ +W_{i1}^{T}\left[\begin{array}{cc} F^{T}\left(jh\right) & 0\\ 0 & F^{T}\left(jh\right) \end{array}\right]Y_{i1}^{T}+Y_{i1}^{}\left[\begin{array}{cc} F\left(jh\right) & 0\\ 0 & F\left(jh\right) \end{array}\right]W_{i1}^{}<0 \end{array}$$

where

$$\begin{array}{l} W_{i1}^{T}=\left[\begin{array}{ccccccc} \sqrt{8}D_{f_{i}}^{T} & 0 & 0 & 0 & 0 & 0 & 0\\ 0 & \sqrt{8}D_{\Theta_{i}}^{T} & 0 & 0 & 0 & 0 & 0 \end{array}\right]\\ Y_{i1}^{T}={\left[\begin{array}{ccccccc} 0 & 0 & 0 & E^{T}{\bar{B}}_{i}^{T}P_{i} & 0 & 0 & 0\\ 0 & 0 & 0 & 0 & E^{T}{\bar{B}}_{i}^{T}P_{i} & 0 & 0 \end{array}\right]}^{T} \end{array}$$

According to the **Lemma 2**, we can get:

(75) $$W_{i1}^{T}F^{T}\left(jh\right)Y_{i1}^{T}+Y_{i1}^{}F\left(jh\right)W_{i1}^{}<\varpi_{i1}^{-1}Y_{i1}^{T}Y_{i1}^{}+\varpi_{i1}W_{i1}^{}W_{i1}^{T}$$

Then, the following inequalities (76) and (77) can ensure that the (75) is true

(76) $$\begin{array}{c} {}[\begin{array}{ccc} \Xi_{i3} & 2{ϒ}_{i}^{T}P_{i} & \sqrt{8}{\left({ϒ}_{i}{\bar{A}}_{i}\right)}^{T}P_{i}\\ 2{ϒ}_{i}P_{i} & -P_{i} & 0\\ \sqrt{8}P_{i}{ϒ}_{i}{\bar{A}}_{i} & 0 & -P_{i}\\ \sqrt{8}P_{i}{ϒ}_{i}{\bar{B}}_{i}EF\left(jh\right)D_{f_{i}}^{} & 0 & 0\\ 0 & \sqrt{8}P_{i}{ϒ}_{i}{\bar{B}}_{i}EF\left(jh\right)D_{\Theta_{i}}^{} & 0 \end{array}\\ \begin{array}{cc} \sqrt{8}D_{f_{i}}^{T}F^{T}\left(jh\right)E^{T}{\bar{B}}_{i}^{T}{ϒ}_{i}^{T}P_{i} & 0\\ 0 & \sqrt{8}D_{\Theta_{i}}^{T}F^{T}\left(jh\right)E^{T}{\bar{B}}_{i}^{T}{ϒ}_{i}^{T}P\\ 0 & 0\\ -P_{i} & 0\\ 0 & -P_{i} \end{array}]<0 \end{array}$$

(77) $$-P_{i}+\varpi_{i1}^{-1}P_{i}{\bar{B}}_{i}EE^{T}{\bar{B}}_{i}^{T}P_{i}<0$$

where $\Xi_{i3}=8{\bar{A}}_{i}^{T}P_{i}{\bar{A}}_{i}-P_{i}+8\varpi_{i1}\left(D_{f_{i}}^{T}D_{f_{i}}^{}+D_{\Theta_{i}}^{T}D_{\Theta_{i}}^{}\right)$.

Similarly, the inequality (76) is equivalent to

(78) $$\begin{array}{l} W_{i2}^{T}\left[\begin{array}{cc} F^{T}\left(jh\right) & 0\\ 0 & F^{T}\left(jh\right) \end{array}\right]Y_{i2}^{T}+Y_{i2}^{}\left[\begin{array}{cc} F\left(jh\right) & 0\\ 0 & F\left(jh\right) \end{array}\right]W_{i2}^{}\\ +[\begin{array}{ccc} \Xi_{i3} & 2{ϒ}_{i}^{T}P_{i} & \sqrt{8}{\left({ϒ}_{i}{\bar{A}}_{i}\right)}^{T}P_{i}\\ 2{ϒ}_{i}P_{i} & -P_{i} & 0\\ \sqrt{8}P_{i}{ϒ}_{i}{\bar{A}}_{i} & 0 & -P_{i}\\ 0 & 0 & 0\\ 0 & 0 & 0 \end{array}\begin{array}{cc} 0 & 0\\ 0 & 0\\ 0 & 0\\ -P_{i} & 0\\ 0 & -P_{i} \end{array}]<0 \end{array}$$

where

$$W_{i2}^{T}=\left[\begin{array}{ccccc} \sqrt{8}D_{f_{i}}^{T} & 0 & 0 & 0 & 0\\ 0 & \sqrt{8}D_{\Theta_{i}}^{T} & 0 & 0 & 0 \end{array}\right]$$

$$Y_{i2}^{T}={\left[\begin{array}{ccccc} 0 & 0 & 0 & E^{T}{\bar{B}}_{i}^{T}{ϒ}_{i}^{T}P_{i} & 0\\ 0 & 0 & 0 & 0 & E^{T}{\bar{B}}_{i}^{T}{ϒ}_{i}^{T}P_{i} \end{array}\right]}^{T}$$

Then the following inequalities (79) and (80) can guarantee the existence of the (78):

(79) $$\left[\begin{array}{ccc} \Xi_{i} & 2{ϒ}_{i}^{T}P_{i} & \sqrt{8}{\left({ϒ}_{i}{\bar{A}}_{i}\right)}^{T}P_{i}\\ 2{ϒ}_{i}P_{i} & -P_{i} & 0\\ \sqrt{8}P_{i}{ϒ}_{i}{\bar{A}}_{i} & 0 & -P_{i} \end{array}\right]<0$$

(80) $$-P_{i}+\varpi_{i2}^{-1}P_{i}{ϒ}_{i}^{}{\bar{B}}_{i}EE^{T}{\bar{B}}_{i}^{T}{ϒ}_{i}^{T}P_{i}<0$$

where $\Xi_{i}=8{\bar{A}}_{i}^{T}P_{i}{\bar{A}}_{i}-P_{i}+8\varpi_{i1}\left(D_{f_{i}}^{T}D_{f_{i}}^{}+D_{\Theta_{i}}^{T}D_{\Theta_{i}}^{}\right)+8\varpi_{i2}\left(D_{f_{i}}^{T}D_{f_{i}}^{}+D_{\Theta_{i}}^{T}D_{\Theta_{i}}^{}\right)$.

Therefore, according to the **Lemma 4**, if LMI inequalities (58)–(60) are true, then $\Sigma_{i}<0$. Therefore, $\Delta V_{i}\left(jh\right)<0$. According to Lyapunov stability theory, the sliding mode dynamic system (57) is asymptotically stable. The proof process is over. 

### 3.3. Discrete Sliding Mode Controller

This is an example of an equation: The control objective of this paper is that the system trajectory tracking error from any initial state can arrive at a switching surface $s_{i}\left(jh\right)=0$ and reach the origin along the sliding surface. So, define a switching belt encircling sliding surface as follows:

(81) $$s_{i}^{\Delta }\left(jh\right)=\left\{s_{i}\left(jh\right)|-\Delta_{ik}\leq s_{ik}\left(jh\right)\leq \Delta_{ik},k=1,2,\ldots ,n\right\}$$

where i=m,s, k denotes the kth coordinate in task space, $s_{i}\left(jh\right)={\left[\begin{array}{ccc} s_{i1}\left(jh\right) & \cdots & s_{in}\left(jh\right) \end{array}\right]}^{T}$, $\Delta_{i}={\left[\begin{array}{ccc} \Delta_{i1} & \cdots & \Delta_{in} \end{array}\right]}^{T}$. The $2\Delta_{i}$ is the width of the switching belt. In order to reduce chattering and improve dynamic quality in the sliding stage, a new reaching law, which consists of the index term $\Phi_{i}\left(jh\right)$ and the convergence parameter $p_{i}>0\left(1-p_{i}h<1\right)$ is designed by

(82) $$s_{i}\left(jh+h\right)=\left(1-p_{i}h\right)\Phi_{i}\left(jh\right)s_{i}\left(jh\right)-\frac{\lambda_{i}}{\Phi_{i}\left(jh\right)}\tanh\left(\frac{s_{i}\left(jh\right)}{\sigma_{i}}\right)$$

where $\lambda_{i}$, i=m,s, is a switching gain, $\sigma_{i}>0$, and

(83) $$\begin{array}{c} \Phi_{i}\left(jh\right)=\delta_{i}+\left(1-\delta_{i}\right)e^{-\varphi_{i}{\left\|s_{i}\left(jh\right)\right\|}^{\gamma_{i}}}\\ \{\begin{array}{c} 0<\delta_{i}<1,\;\varphi_{i}>0\\ \gamma_{i}>0,\;and\;\gamma_{i}\in N \end{array} \end{array}$$

**Remark** **2.** *In practical applications, the tanh function is advocated to replace the sign function in order to reduce chattering, and a mass of experimental data also verifies its effectiveness. However, no one has theoretically analyzed the feasibility for the tanh function. This paper will make an attempt in this area. Theoretical analysis will be presented in **Theorem 3**. According to expression (83), a conclusion can be drawn that if the system tracking error is far away from the discrete sliding surface, $\Phi_{i}\left(jh\right)$ tends to $\delta_{i}$. Thus $\left(1-p_{i}h\right)\Phi_{i}\left(jh\right)$ tends to $\left(1-p_{i}h\right)\delta_{i}$, which is less than $\left(1-p_{i}h\right)$, and $\frac{\lambda_{i}}{\Phi_{i}\left(jh\right)}$ tends to $\frac{\lambda_{i}}{\delta_{i}}$, which is greater than $\lambda_{i}$. On the contrary, when the system tracking error is close to the discrete sliding surface, $\Phi_{i}\left(jh\right)$ tends to 1. Thus $\left(1-p_{i}h\right)\Phi_{i}\left(jh\right)$ tends to $\left(1-p_{i}h\right)$, and $\frac{\lambda_{i}}{\Phi_{i}\left(jh\right)}$ tends to $\lambda_{i}$. Obviously, the index term $\Phi_{i}\left(jh\right)$ is positive, therefore the stability of the nonlinear bilateral teleoperation system will be unaffected.*

With consideration of the system (8) and the new reaching law (82), the proposed DSMC controller for the nonlinear bilateral teleoperation system can be given as

(84) $$\begin{array}{ll} u_{i}\left(jh\right)= & -{\left(\Lambda_{i}{\bar{B}}_{i}H_{i}\right)}^{-1}(\Lambda_{i}{\bar{A}}_{i}X_{i}\left(jh\right)+\Lambda_{i}{\bar{B}}_{i}f_{i}\left(jh\right)-\Lambda_{i}X_{di}\left(jh+h\right)\\ & -\left(1-p_{i}h\right)\Phi_{i}\left(jh\right)s_{i}\left(jh\right)+\frac{\lambda_{i}}{\Phi_{i}\left(jh\right)}\tanh\left(\frac{s_{i}\left(jh\right)}{\sigma_{i}}\right))-M_{oi}{\hat{\Theta }}_{i}\left(jh\right) \end{array}$$

**Figure 1:**
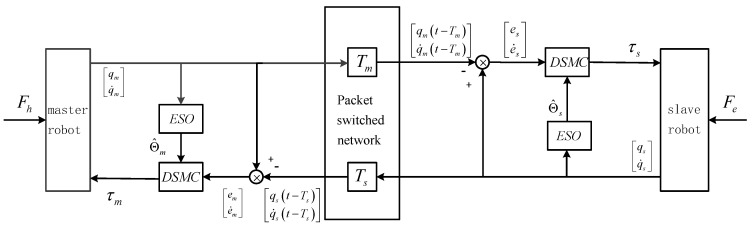
Control block diagram of teleoperation system with adaptive extended state observer (AESO)-based discrete sliding mode control scheme.

The control block diagram of nonlinear bilateral teleoperation system with controller (86) is displayed in Figure 1.

For the design of the discrete sliding mode controller, the following lemma and assumption will be utilized.

**Lemma** **5** **([34]).** *For*∀x∈R*,*$\left\|x\right\|-x\tanh\left(\frac{x}{\delta }\right)\leq 0.2785\delta$*, where*δ>0∈R*.*

**Assumption** **6.** *According to the **Theory 1**, the disturbance* *estimation error* $\Theta_{i}\left(jh\right)-{\hat{\Theta }}_{i}\left(jh\right)$ *is bounded and converges to a very small range. Therefore, it is reasonable to assume that:*

(85) $$\left\|\varsigma_{ik}\left(jh\right)\right\|\leq \varsigma_{ik}$$

*where i=m,s, k=1,2,…,n, $\varsigma_{i}\left(jh\right)=\Lambda_{i}{\bar{B}}_{i}\left(\Theta_{i}\left(jh\right)-{\hat{\Theta }}_{i}\left(jh\right)\right)={\left[\begin{array}{ccc} \varsigma_{i1}\left(jh\right) & \cdots & \varsigma_{in}\left(jh\right) \end{array}\right]}^{T}$, $\varsigma_{i}={\left[\begin{array}{ccc} \varsigma_{i1} & \cdots & \varsigma_{in} \end{array}\right]}^{T}$, and $\varsigma_{ik}$ is the positive constant and denotes the upper bound of $\varsigma_{ik}\left(jh\right)$.*

**Theorem** **3.** *For the discrete nonlinear bilateral teleoperation system (89) based on the controller (84), under the **Assumption6**, if the following condition is maintained:*

(86) $$\varsigma_{ik}\leq \lambda_{i}$$

*(a). The system trajectory tracking error from any initial state will enter this switching belt $s_{i}^{\Delta }\left(jh\right)$ of DSMC defined by*

(87) $$s_{i}^{\Delta }\left(jh\right)=\left\{s_{i}\left(jh\right)|\left\|s_{ik}\left(jh\right)\right\|\leq \Delta_{ik}=\frac{0.2785\delta_{i}\lambda_{i}}{\lambda_{i}-\varsigma_{ik}\Phi_{i}\left(jh\right)},k=1,2,\ldots ,n\right\}$$

*(b). Once the system trajectory tracking errors enter this switching belt $s_{i}^{\Delta }\left(jh\right)$, they cannot escape from it.*

In this paper, because the coupling relationship between the states of nonlinear bilateral teleoperation system is compensated as the uncertainty during the AESO design phase, with regard to ∀k, the controllers are independent on each other and then the stability analysis can be demonstrated in the same way. Hereinafter, the stability analysis is discussed for only one.

**Proof.**  In this proof, two cases will be considered, namely, the positive and negative values of $s_{ik}\left(jh\right)$. The equivalent form of **Theorem 3** is represented as follows:

(88) $$-\frac{0.2785\delta_{i}\lambda_{i}}{\lambda_{i}-\varsigma_{ik}\Phi_{i}\left(jh\right)}<s_{ik}\left(jh\right)<\frac{0.2785\delta_{i}\lambda_{i}}{\lambda_{i}-\varsigma_{ik}\Phi_{i}\left(jh\right)}$$

where i=m,s, k=1,2,…,n.

**(a). Case 1:** If $s_{ik}\left(jh\right)>0$, due to $0<\Phi_{i}\left(jh\right)<1$, it can be obtained from (82) and (86) that:

(89) $$\begin{array}{ll} s_{ik}\left(jh+h\right) & =\left(1-p_{i}h\right)\Phi_{i}\left(jh\right)s_{ik}\left(jh\right)-\frac{\lambda_{i}}{\Phi_{i}\left(jh\right)}\tanh\left(\frac{s_{ik}\left(jh\right)}{\sigma_{i}}\right)+\varsigma_{ik}\left(jh\right)\\ & \leq s_{ik}\left(jh\right)-\frac{\lambda_{i}}{\Phi_{i}\left(jh\right)}\tanh\left(\frac{s_{ik}\left(jh\right)}{\sigma_{i}}\right)+\varsigma_{ik} \end{array}$$

According to the **Lemma 5**, then

(90) $$\begin{array}{ll} s_{ik}\left(jh+h\right) & \leq s_{ik}\left(jh\right)-\frac{\lambda_{i}}{\Phi_{i}\left(jh\right)}\left(1-\frac{0.2785\delta_{i}}{s_{ik}\left(jh\right)}\right)+\varsigma_{ik}\\ & \leq s_{ik}\left(jh\right) \end{array}$$

Therefore, the sequence $\left\{s_{ik}\left(jh\right)\right\}$ is strictly monotonously decreasing when $s_{ik}\left(jh\right)>0$. It is concluded that there must exist a positive integer $j=j^{*}$ so that the following inequality (91) holds:

(91) $$\begin{array}{ll} s_{ik}\left(j^{*}h+h\right) & =\left(1-p_{i}h\right)\Phi_{i}\left(j^{*}h\right)s_{ik}\left(j^{*}h\right)-\frac{\lambda_{i}}{\Phi_{i}\left(j^{*}h\right)}\tanh\left(\frac{s_{ik}\left(j^{*}h\right)}{\sigma_{i}}\right)+\varsigma_{ik}\left(j^{*}h\right)\\ & \leq \frac{0.2785\delta_{i}\lambda_{i}}{\lambda_{i}-\varsigma_{ik}\Phi_{i}\left(j^{*}h\right)} \end{array}$$

when $j\geq j^{*}$, the system trajectory tracking error enters the switching belt $s_{i}^{\Delta }\left(jh\right)$ of DSMC.

**Case 2:** If $s_{ik}\left(jh\right)<0$, due to $0<\Phi_{i}\left(jh\right)<1$ and the condition (86), it can be obtained that

(92) $$\begin{array}{l} s_{ik}\left(jh+h\right)=\left(1-p_{i}h\right)\Phi_{i}\left(jh\right)s_{ik}\left(jh\right)-\frac{\lambda_{i}}{\Phi_{i}\left(jh\right)}\tanh\left(\frac{s_{ik}\left(jh\right)}{\sigma_{i}}\right)+\varsigma_{ik}\left(jh\right)\\ \;\geq s_{ik}\left(jh\right)-\frac{\lambda_{i}}{\Phi_{i}\left(jh\right)}\tanh\left(\frac{s_{ik}\left(jh\right)}{\sigma_{i}}\right)+\varsigma_{ik}\\ \;\geq s_{ik}\left(jh\right)-\frac{\lambda_{i}}{\Phi_{i}\left(jh\right)}\left(1-\frac{0.2785\delta_{i}}{s_{ik}\left(jh\right)}\right)+\varsigma_{ik}\\ \;\geq s_{ik}\left(jh\right) \end{array}$$

Therefore, the sequence $\left\{s_{ik}\left(jh\right)\right\}$ is strictly monotonously increasing when $s_{ik}\left(jh\right)<0$. It is concluded that there must exist a positive integer $j=j^{*}$ so that the following inequality (93) holds:

(93) $$\begin{array}{ll} s_{ik}\left(j^{*}h+h\right) & =\left(1-p_{i}h\right)\Phi_{i}\left(j^{*}h\right)s_{ik}\left(j^{*}h\right)-\frac{\lambda_{i}}{\Phi_{i}\left(j^{*}h\right)}\tanh\left(\frac{s_{ik}\left(j^{*}h\right)}{\sigma_{i}}\right)+\varsigma_{ik}\left(j^{*}h\right)\\ & >-\frac{0.2785\delta_{i}\lambda_{i}}{\lambda_{i}-\varsigma_{ik}\Phi_{i}\left(j^{*}h\right)} \end{array}$$

when $j\geq j^{*}$, the system trajectory tracking error enters the switching belt $s_{i}^{\Delta }\left(jh\right)$ of DSMC.

Therefore, in view of (91) and (93), it can be concluded that if $s_{ik}\left(jh\right)$ lies outside the switching belt $s_{i}^{\Delta }\left(jh\right)$ defined by (87), then the system trajectory tracking error from any initial state will enter into this switching belt of DSMC.

**(b). Case 1:** When the $s_{ik}\left(jh\right)$ enters the switching belt $s_{i}^{\Delta }\left(jh\right)$, namely $0<s_{ik}\left(jh\right)<\frac{0.2785\delta_{i}\lambda_{i}}{\lambda_{i}-\varsigma_{ik}\Phi_{i}\left(jh\right)}$, then:

(94) $$\begin{array}{ll} s_{ik}\left(jh+h\right) & =\left(1-p_{i}h\right)\Phi_{i}\left(jh\right)s_{ik}\left(jh\right)-\frac{\lambda_{i}}{\Phi_{i}\left(jh\right)}\tanh\left(\frac{s_{ik}\left(jh\right)}{\sigma_{i}}\right)+\varsigma_{ik}\left(jh\right)\\ & \geq -\frac{\lambda_{i}}{\Phi_{i}\left(jh\right)}\tanh\left(\frac{s_{ik}\left(jh\right)}{\sigma_{i}}\right)+\varsigma_{ik}\left(jh\right)\\ & \geq -\frac{0.2785\delta_{i}\lambda_{i}}{\lambda_{i}-\varsigma_{ik}\Phi_{i}\left(jh\right)} \end{array}$$

Suppose $s_{ik}\left(jh\right)$ has escaped the switching belt $s_{i}^{\Delta }\left(jh\right)$ again, namely, $s_{ik}\left(jh+h\right)\geq \frac{0.2785\delta_{i}\lambda_{i}}{\lambda_{i}-\varsigma_{ik}\Phi_{i}\left(jh\right)}\geq s_{ik}\left(jh\right)$, then

(95) $$\begin{array}{ll} s_{ik}\left(jh+h\right) & =\left(1-p_{i}h\right)\Phi_{i}\left(jh\right)s_{ik}\left(jh\right)-\frac{\lambda_{i}}{\Phi_{i}\left(jh\right)}\tanh\left(\frac{s_{ik}\left(jh\right)}{\sigma_{i}}\right)+\varsigma_{ik}\left(jh\right)\\ & <s_{ik}\left(jh\right)-\frac{\lambda_{i}}{\Phi_{i}\left(jh\right)}\tanh\left(\frac{s_{ik}\left(jh\right)}{\sigma_{i}}\right)+\varsigma_{ik}\left(jh\right)\\ & <\frac{0.2785\delta_{i}\lambda_{i}}{\lambda_{i}-\varsigma_{ik}\Phi_{i}\left(jh\right)} \end{array}$$

However, the result is inconsistent with the hypothesis $s_{ik}\left(jh+h\right)\geq s_{ik}\left(jh\right)$, so that the hypothesis is not set up. Hence,

(96) $$s_{ik}\left(jh+h\right)<\frac{0.2785\delta_{i}\lambda_{i}}{\lambda_{i}-\varsigma_{ik}\Phi_{i}\left(jh\right)}$$

**Case 2:** When the $s_{ik}\left(jh\right)$ enters the switching belt $s_{i}^{\Delta }\left(jh\right)$ defined by (87), namely, $-\frac{0.2785\delta_{i}\lambda_{i}}{\lambda_{i}-\varsigma_{ik}\Phi_{i}\left(jh\right)}<s_{ik}\left(jh\right)<0$, then

(97) $$\begin{array}{ll} s_{ik}\left(jh+h\right) & =\left(1-p_{i}h\right)\Phi_{i}\left(jh\right)s_{ik}\left(jh\right)-\frac{\lambda_{i}}{\Phi_{i}\left(jh\right)}\tanh\left(\frac{s_{ik}\left(jh\right)}{\sigma_{i}}\right)+\varsigma_{ik}\left(jh\right)\\ & <-\frac{\lambda_{i}}{\Phi_{i}\left(jh\right)}\tanh\left(\frac{s_{ik}\left(jh\right)}{\sigma_{i}}\right)+\varsigma_{ik}\left(jh\right)\\ & <\frac{0.2785\delta_{i}\lambda_{i}}{\lambda_{i}-\varsigma_{ik}\Phi_{i}\left(jh\right)} \end{array}$$

Suppose $s_{ik}\left(jh\right)$ has escaped the switching belt $s_{i}^{\Delta }\left(jh\right)$ again, namely, $s_{ik}\left(jh+h\right)\leq -\frac{0.2785\delta_{i}\lambda_{i}}{\lambda_{i}-\varsigma_{ik}\Phi_{i}\left(jh\right)}\leq s_{ik}\left(jh\right)$, then

(98) $$\begin{array}{ll} s_{ik}\left(jh+h\right) & =\left(1-p_{i}h\right)\Phi_{i}\left(jh\right)s_{ik}\left(jh\right)-\frac{\lambda_{i}}{\Phi_{i}\left(jh\right)}\tanh\left(\frac{s_{ik}\left(jh\right)}{\sigma_{i}}\right)+\varsigma_{ik}\left(jh\right)\\ & >s_{ik}\left(jh\right)-\frac{\lambda_{i}}{\Phi_{i}\left(jh\right)}\tanh\left(\frac{s_{ik}\left(jh\right)}{\sigma_{i}}\right)+\varsigma_{ik}\left(jh\right)\\ & >-\frac{0.2785\delta_{i}\lambda_{i}}{\lambda_{i}-\varsigma_{ik}\Phi_{i}\left(jh\right)} \end{array}$$

However, the result is inconsistent with the hypothesis $s_{ik}\left(jh+h\right)\leq s_{ik}\left(jh\right)$ so that the hypothesis is not set up. Hence,

(99) $$s_{ik}\left(jh+h\right)\geq -\frac{0.2785\delta_{i}\lambda_{i}}{\lambda_{i}-\varsigma_{ik}\Phi_{i}\left(jh\right)}$$

In short, $s_{ik}\left(jh\right)\in \left[\frac{-0.2785\delta_{i}\lambda_{i}}{\lambda_{i}-\varsigma_{ik}\Phi_{i}\left(jh\right)},\frac{0.2785\delta_{i}\lambda_{i}}{\lambda_{i}-\varsigma_{ik}\Phi_{i}\left(jh\right)}\right]$, when $s_{ik}\left(jh+h\right)\in \left[\frac{-0.2785\delta_{i}\lambda_{i}}{\lambda_{i}-\varsigma_{ik}\Phi_{i}\left(jh\right)},\frac{0.2785\delta_{i}\lambda_{i}}{\lambda_{i}-\varsigma_{ik}\Phi_{i}\left(jh\right)}\right]$. That is to say, once the system trajectory tracking error enters this switching belt $s_{i}^{\Delta }\left(jh\right)$, they cannot escape from it. □

## 4. Simulation and Experiment

In this section, the simulated analyses and experimental results on the strength of the proposed control strategy are presented, with the purpose of verifying the effectiveness of the discrete SMC algorithm and the superiority of its control performance. Firstly, the simulations are implemented on two three-degree-of-freedom robot arms

(100) $$\left\{ \begin{array}{l} M_{q_{m}}(q_{m}){\ddot{q}}_{m}+C_{q_{m}}(q_{m},{\dot{q}}_{m}){\dot{q}}_{m}+g_{q_{m}}(q_{m})+f_{q_{m}}({\dot{q}}_{m})+B_{q_{m}}(q_{m})=\tau_{m}+J_{m}^{T}\left(q_{m}\right)F_{h}\\ M_{q_{s}}(q_{s}){\ddot{q}}_{s}+C_{q_{s}}(q_{s},{\dot{q}}_{s}){\dot{q}}_{s}+g_{q_{s}}(q_{s})+f_{q_{s}}({\dot{q}}_{s})+B_{q_{s}}(q_{s})=\tau_{s}-J_{s}^{T}\left(q_{s}\right)F_{e} \end{array}\right.$$

The kinematic relationship between task space and joint space of the nonlinear bilateral teleoperation system is given by

(101) $$\begin{array}{l} \chi_{i1}=l_{i1}\cos(q_{i1})+l_{i2}\cos(q_{i1}+q_{i2})+l_{i3}\cos(q_{i1}+q_{i2}+q_{i3})\\ \chi_{i2}=l_{i1}\sin(q_{i1})+l_{i2}\sin(q_{i1}+q_{i2})+l_{i3}\sin(q_{i1}+q_{i2}+q_{i3})\\ \chi_{i3}=q_{i1}+q_{i2}+q_{i3} \end{array}$$

where i=m,s, $l_{i1},l_{i2},l_{i3}$ represent the link lengths and $q_{i1},q_{i2},q_{i3}$ represent the joint angles of the robot arms.

**Figure 2:**
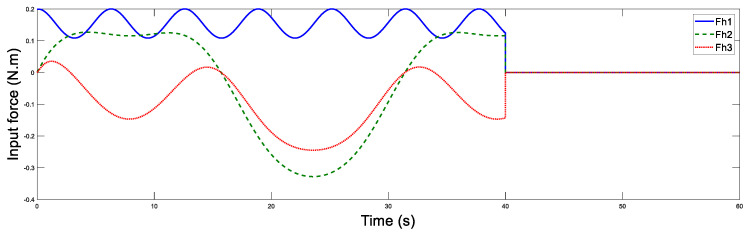
Human force of the master.

For the simulation, the relevant parameter values are set as $l_{i1}=2.05$, $l_{i2}=2.05$, $l_{i3}=0.5$, i=m,s. The human force $F_{h}$ is imposed on the master robot, which is depicted in Figure 2. While in the slave site, the force is kept to zero. The simulation results are utilized to prove the following: (1) When the master robot moves, does the slave robot follow the master? (2) When the human-input force vanishes, does the tracking error between the master and slave vanish as well? The aim of (1) and (2) is to present the stability of the nonlinear bilateral teleoperation system.

Now, we evaluate the validity of the proposed control strategy for the nonlinear bilateral teleoperation system (100) with existence of both time delays and flexible friction forces. First, time delays are adjusted to $T_{m}=T_{s}=300\;\mathrm{ms}$. The sampling period is h=0.001 s, and parameters of adaptive ESO are chosen as

$$eig\left({\bar{R}}_{m}\right)=eig\left({\bar{R}}_{s}\right)={\left[\begin{array}{ccc} 0.002^{2} & 0.002^{2} & 0.002^{2} \end{array}\right]}^{T}$$

$$eig\left({\bar{Q}}_{m}\right)=eig\left({\bar{Q}}_{s}\right)={\left[\begin{array}{ccc} 0.1^{2} & 0.1^{2} & 0.1^{2} \end{array}\right]}^{T}$$

$$eig\left({\bar{Q}}_{m}\right)=eig\left({\bar{Q}}_{s}\right)={\left[\begin{array}{ccccccccc} 0.005^{2} & 0.005^{2} & 0.005^{2} & 0.05^{2} & 0.05^{2} & 0.05^{2} & 0.04^{2} & 0.04^{2} & 0.04^{2} \end{array}\right]}^{T}$$

Parameters of discrete-time sliding mode surface are set as $\lambda_{m}=\lambda_{s}=0.15$, $\delta_{m}=\delta_{s}=0.25$, $\varphi_{m}=\varphi_{s}=20$, $\gamma_{m}=\gamma_{s}=10$, $\Lambda_{m}=\Lambda_{s}=\left[\begin{array}{cccccc} 0.3 & 0 & 0 & 0.05 & 0 & 0\\ 0 & 0.2 & 0 & 0 & 0.02 & 0\\ 0 & 0 & 0.2 & 0 & 0 & 0.02 \end{array}\right]$, $\sigma_{m}=\sigma_{s}=1$, $p_{m}=p_{s}=350$.

**Figure 3:**
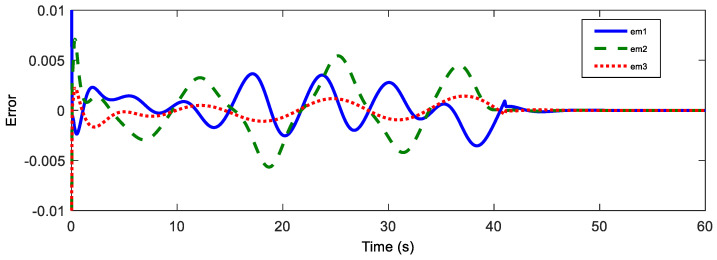
Position estimation error of the master.

**Figure 4:**
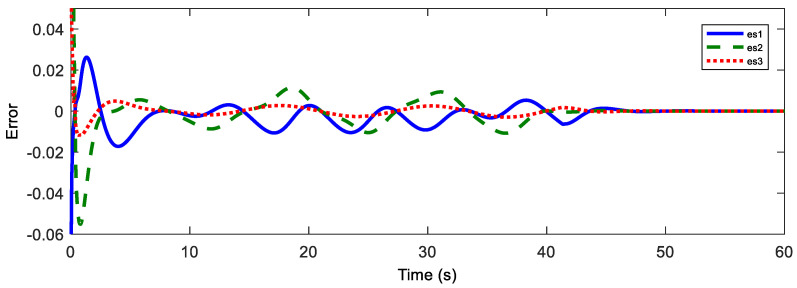
Position estimation error of the master.

Afterwards, the trajectory estimation errors of master and slave robots are exhibited in Figure 3 and Figure 4, respectively. 

**Figure 5:**
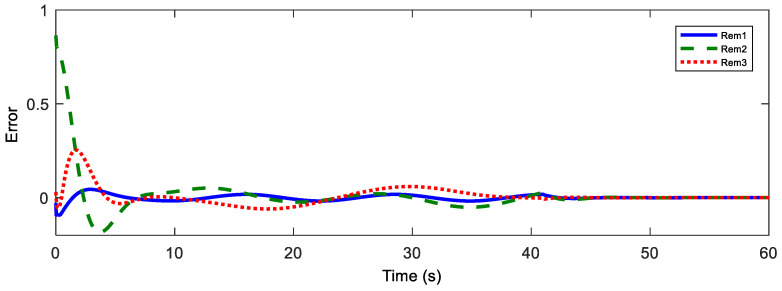
Disturbance estimation errors of the master.

**Figure 6:**
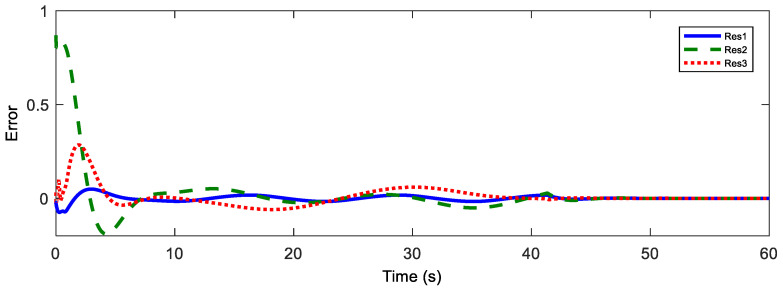
Disturbance estimation errors of the slave.

**Figure 7:**
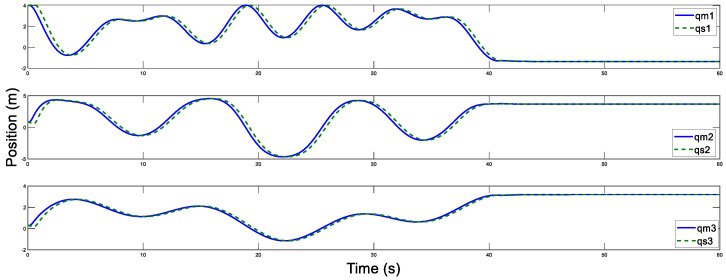
Position tracking motion for the master and slave.

**Figure 8:**
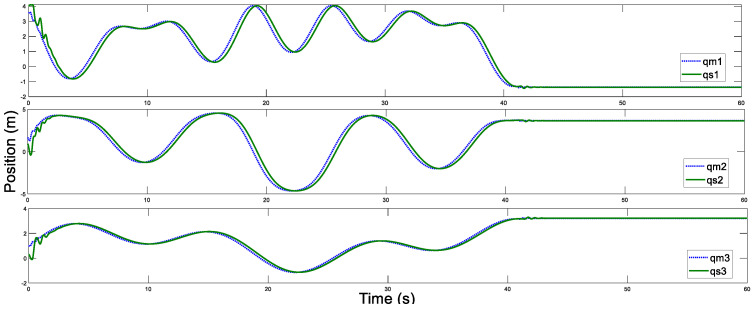
Position tracking motion for master and slave with PD controller.

The disturbance estimation errors of the master and the slave are displayed in Figure 5 and Figure 6. As shown, estimation errors of both the motion trajectory and the disturbance converge to a very small range, and then approach rapidly to zero after $F_{h}=0$. Therefore, accurate estimation of total system disturbances is provided by AESO. In order to show the superiority of the proposed controller, PD controller (Proportional derivative controller) is taken as the comparison term. Now, the motion trajectories of the master and the slave end effectors with proposed DSMC are shown in Figure 7. Obviously, the slave can accurately reproduce the trajectory of the master and the prominent synchronization performance is completed. On the contrary, in Figure 8, at the beginning of the movement, the chattering is quite serious, and the position tracking effect of the master robot and the slave robot is poor.

**Figure 9:**
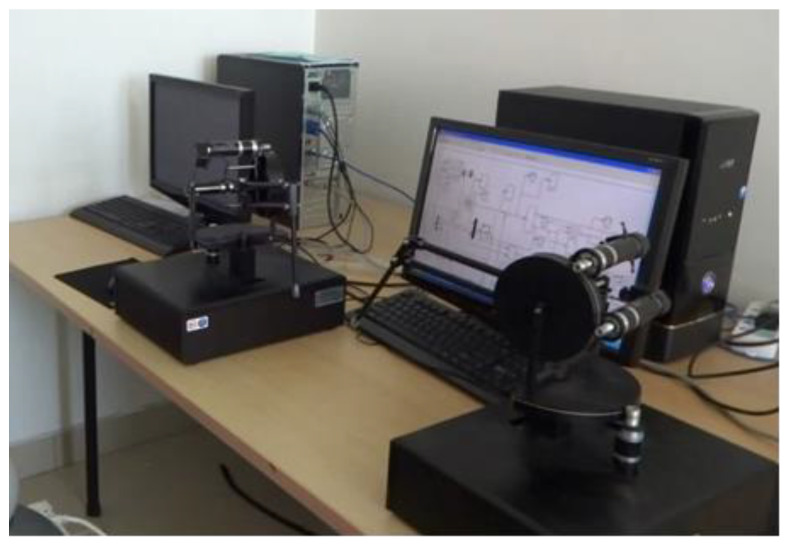
The nonlinear bilateral teleoperation experiment platform.

To further prove the superiority of the controller designed in this paper, the control algorithm is implemented on the teleoperation experiment platform built in the laboratory. The nonlinear bilateral teleoperation system model includes a couple of Phantom Premium 1.5A robot arms (SensAble Technologies, Inc.) to be performed, which is shown in Figure 9. In the experiment, the sampling period is h=0.002 s, and the parameters of the discrete sliding mode control algorithm are selected as $\lambda_{m}=\lambda_{s}=0.4$, $\delta_{m}=\delta_{s}=0.25$, $\varphi_{m}=\varphi_{s}=20$, $\gamma_{m}=\gamma_{s}=10$, $\Lambda_{m}=\Lambda_{s}=\left[\begin{array}{cccccc} 0.3 & 0 & 0 & 0.08 & 0 & 0\\ 0 & 0.3 & 0 & 0 & 0.05 & 0\\ 0 & 0 & 0.3 & 0 & 0 & 0.05 \end{array}\right]$, $\sigma_{m}=\sigma_{s}=1$, $p_{m}=p_{s}=350$.

Then the parameters of adaptive ESO are chosen as

$$eig\left({\bar{R}}_{m}\right)=eig\left({\bar{R}}_{s}\right)={\left[\begin{array}{ccc} 0.002^{2} & 0.002^{2} & 0.002^{2} \end{array}\right]}^{T}$$

$$eig\left({\bar{Q}}_{m}\right)=eig\left({\bar{Q}}_{s}\right)={\left[\begin{array}{ccc} 0.1^{2} & 0.1^{2} & 0.1^{2} \end{array}\right]}^{T}$$

$$\begin{array}{c} eig\left({\bar{Q}}_{m}\right)=eig\left({\bar{Q}}_{s}\right)=[\begin{array}{ccccc} 6\times 10^{-18} & 6\times 10^{-18} & 6\times 10^{-18} & 0.0245^{2} & 0.0245^{2} \end{array}\\ {\begin{array}{cccc} 0.0245^{2} & 12.247^{2} & 12.247^{2} & 12.247^{2} \end{array}]}^{T} \end{array}$$

**Figure 10:**
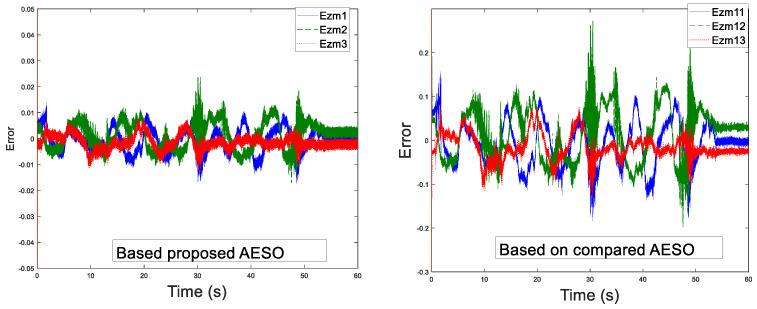
Master position estimation errors with $T_{m}$ = 200 ms.

**Figure 11:**
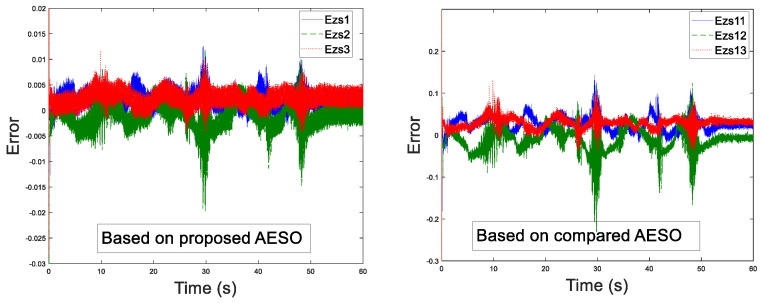
Slave position estimation errors with $T_{s}$ = 200 ms.

**Figure 12:**
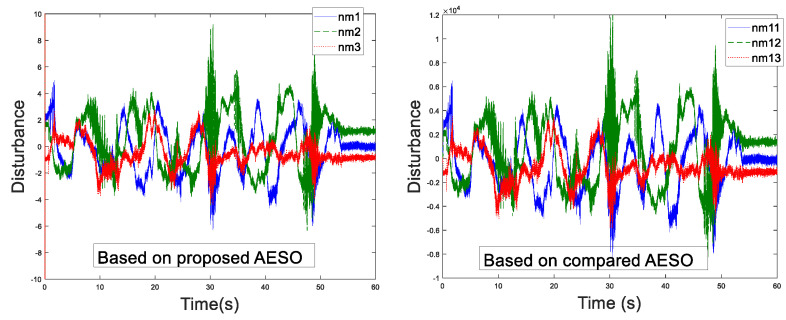
Master disturbance estimation with $T_{m}$ = 200 ms.

**Figure 13:**
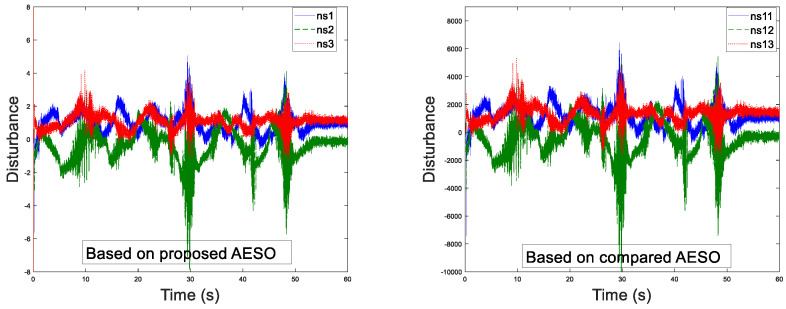
Slave disturbance estimation with $T_{s}$ = 200 ms.

**Figure 14:**
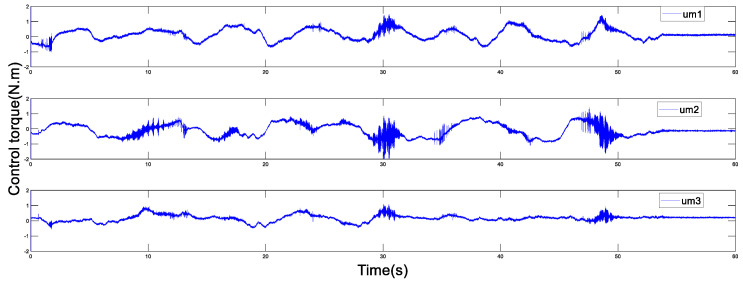
Control torque of the master with $T_{m}=200\;\mathrm{ms}$.

**Figure 15:**
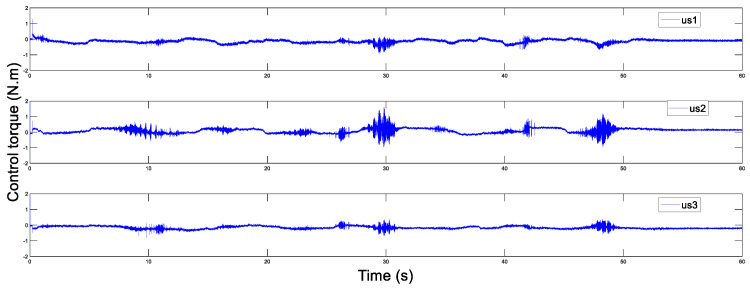
Control torque of the slave with $T_{s}=200\;\mathrm{ms}$.

**Figure 16:**
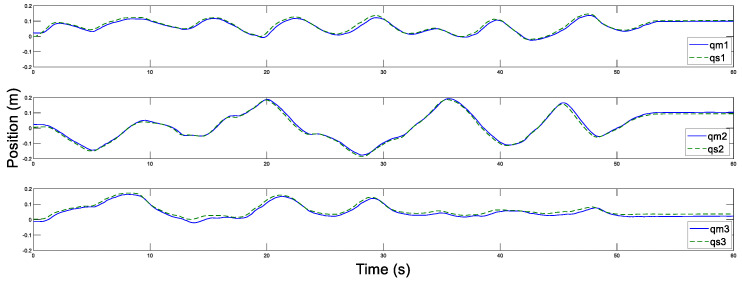
Position tracking motion of the master and the slave with $T_{m}=T_{s}=200\;\mathrm{ms}$.

Before the experiment, the information transmission delay between the master and the slave robots by Simulink module is set to $T_{m}=T_{s}=200\;ms$. With the aim of verifying the performance superiority of the AESO designed in this paper, the contrast experiment between the proposed AESO and another compared one with a constant parameter $\theta_{i}$ is provided. The experiment results are displayed in Figure 10, Figure 11, Figure 12, Figure 13, Figure 14, Figure 15 and Figure 16. As shown in Figure 10, Figure 11, Figure 12 and Figure 13, the motion trajectory estimation errors and the total disturbance estimation of the master and the slave based on this paper and the compared one is shown. It is noticed that the trajectory estimation errors based on this paper converge within 0.02 m, while others based on the compared one converge within 0.3 m. Comparing the total disturbance estimation, the AESO designed in this paper could effectively estimate the total disturbance; whereas, another one based on the compared one brings a hundredfold increase in total disturbance estimation and thus loses the estimation performance.

In addition, Figure 14 and Figure 15 display the control torques of the master and the slave robots, respectively. The motion trajectory tracking between the master and the slave is shown in Figure 16. We can see that the master robot stops moving after 50 s, and then the slave robot also promptly stops moving. Ultimately, the two robots stop in the same position.

**Figure 17:**
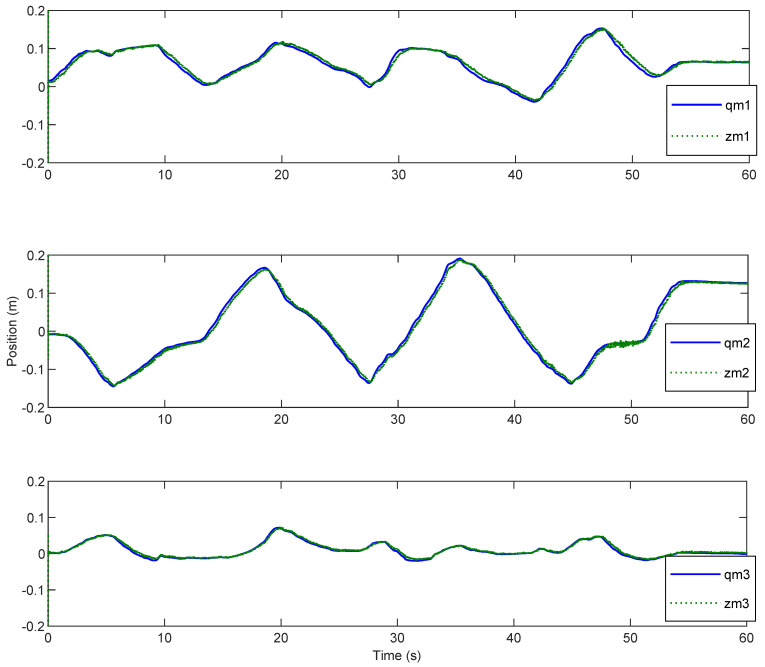
Master position estimation with $T_{m}=500\;\mathrm{ms}$.

**Figure 18:**
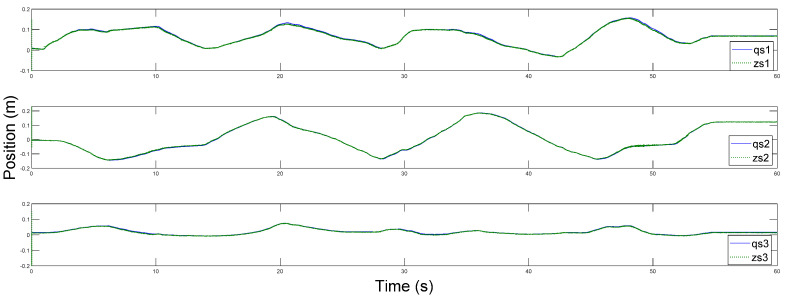
Slave position estimation with $T_{s}=500\;\mathrm{ms}$.

**Figure 19:**
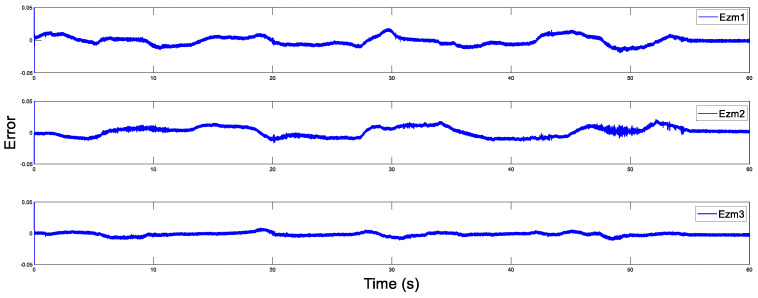
Master position estimation errors with $T_{m}=500\;\mathrm{ms}$.

**Figure 20:**
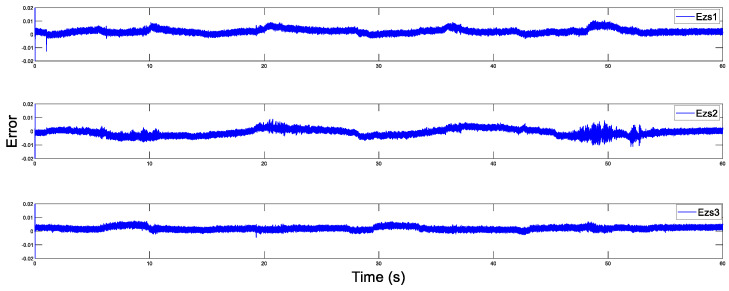
Slave position estimation errors with $T_{s}=500\;\mathrm{ms}$.

**Figure 21:**
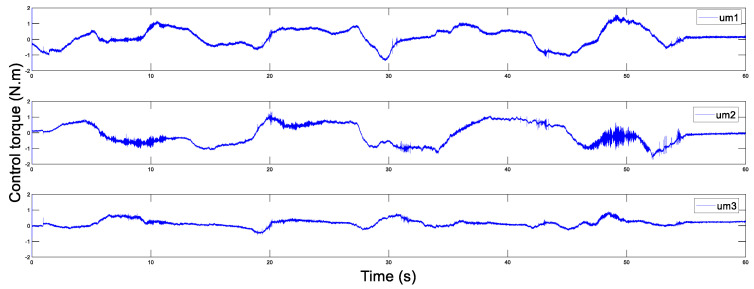
Control torque of the master $T_{m}=500\;\mathrm{ms}$.

**Figure 22:**
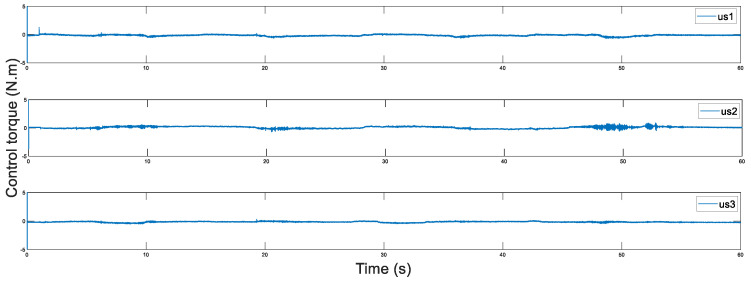
Control torque of the slave with $T_{s}=500\;\mathrm{ms}$.

**Figure 23:**
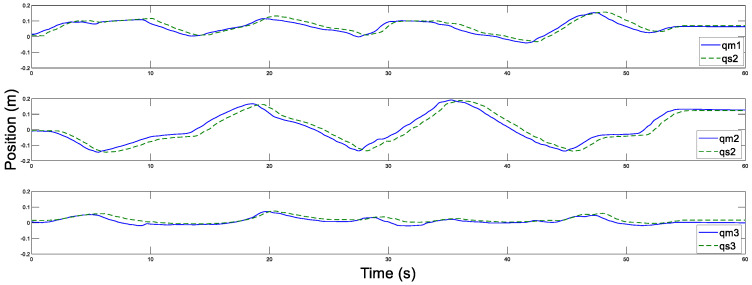
Position tracking motion of the master and the slave with $T_{m}=T_{s}=500\;\mathrm{ms}$.

Next, the communication time delay is increased to $T_{m}=T_{s}=500$ ms. The trajectory estimations and the estimation errors for the master and the slave based on this paper are displayed in Figure 17, Figure 18, Figure 19 and Figure 20. The control torques of the master and the slave robots are shown in Figure 21 and Figure 22, respectively. Furthermore, Figure 23 presents the motion trajectory tracking between the master and the slave robots. From this experiment, although the slave robot responds slowly due to the increased time delay, it still follows the master motion. The experimental results and analyses further validate the superiority and validity of the proposed control strategy in this paper.

## 5. Conclusions

This paper discusses the synchronization control issue for asymmetric bilateral teleoperation systems with time delays. Due to the discrete states caused by unreliable communication networks, new task-space-based discrete sliding mode controllers are designed. The new reaching law of DSMC consisting of the index term and tanh function is developed to reduce the chattering significantly. In addition, the input controllers are formed by undelayed position signals, delayed reference signals, and AESO-based term to compensate the total disturbance associated with the master and slave robots model dynamic. For the sake of stabilizing AESO, a new parameter θ is introduced in the form of a matrix to adjust the corresponding gain for each state separately. It is shown that estimation errors of AESO and tracking errors of teleoperation system are bound in a certain range, which is not affected by time delays and flexible friction forces. Finally, the simulated and experimental results are provided to reveal the validity and superiority of the proposed strategy.

## Nomenclature

${\left\{•\right\}}_{m},{\left\{•\right\}}_{s}$	Subscripts {m,s} denote the master and slave robots, respectively
${\left\{•\right\}}_{s}$	Subscripts s denote the slave robots
$q_{i}$	Joint position of the master (i=m)/slave (i=s) robot in joint space
${\dot{q}}_{i}$	Joint velocity of the master (i=m)/slave (i=s) robot in joint space
${\ddot{q}}_{i}$	Joint acceleration of the master (i=m)/slave (i=s) robot in joint space
$\chi_{i},{\dot{\chi }}_{i},{\ddot{\chi }}_{i}$	Position, velocity, acceleration of the master (i=m)/slave (i=s) task-space end effectors
$M_{q_{i}}(q_{i})$	Symmetric positive definite inertia matrix of the master (i=m)/slave (i=s) robot in joint space
$M_{i}(q_{i})$	Symmetric positive definite inertia matrix of the master (i=m)/slave (i=s) robot in task space
$C_{q_{i}}(q_{i},{\dot{q}}_{i}){\dot{q}}_{i}$	Coriolis/centrifugal matrix of the master (i=m)/slave (i=s) robot in joint space
$C_{i}(q_{i},{\dot{q}}_{i}){\dot{q}}_{i}$	Coriolis/centrifugal matrix of the master (i=m)/slave (i=s) robot in task space
$g_{q_{i}}(q_{i})$	Gravitational torque of the master (i=m)/slave (i=s) robot in joint space
$g_{i}(q_{i})$	Gravitational torque of the master (i=m)/slave (i=s) robot in task space
$f_{q_{i}}({\dot{q}}_{i})$	Viscous friction vector of the master (i=m)/slave (i=s) robot in joint space
$f_{i}({\dot{q}}_{i})$	Viscous friction vector of the master (i=m)/slave (i=s) robot in task space
$B_{q_{i}}(q_{i})$	Disturbance vector of the master (i=m)/slave (i=s) robot in joint space
$B_{i}(q_{i})$	Disturbance vector of the master (i=m)/slave (i=s) robot in task space
$F_{h},F_{e}$	Forces exerted on the end-effectors of the master and slave robots by the human operator and environment in joint space, respectively
$\tau_{i}(q_{i})$	Control torque of the master (i=m) /slave (i=s) robot in joint space
$u_{i}$	Input control torque of the master (i=m)/slave (i=s) robot in task space
$J_{i}\left(q_{i}\right)$	Jacobian matrix of the master (i=m)/slave (i=s) robot
$T_{i}$	Time delays from the master robot to the slave robot and from the slave robot to the master robot in task space, respectively
$X_{i}$	Position and speed state vector set of the master (i=m)/slave (i=s) robot in task space
$X_{di}$	Position tracking in task space
$\Theta_{i}$	Total disturbance vector of the master (i=m)/slave (i=s) robot in task space
A	State coefficient matrix in the system equation
B	Unknown coefficient matrix in the system equation
$\left\{\bar{•}\right\}$	Discrete form of corresponding coefficient matrix
$\left\{\tilde{•}\right\}$	The expanded form of the corresponding coefficient matrix
h	Sampling time of the master (i=m)/slave (i=s) robot in task space
j	Jth sampling moment of the master (i=m)/slave (i=s) robot in task space
R	Real number vector set
$R^{n}$	N-dimensional real number vector set
$R^{n\times n}$	N-row and n-column real matrix set
$R^{n\times 2n}$	N-row and 2n-column real matrix set
I	Identity matrix with appropriate dimensions
${\left\{•\right\}}^{T}$	Superscript T denotes transpose matrix
${\left\{•\right\}}^{-1}$	Superscript -1 denotes inverse matrix
$\left\{\hat{•}\right\}$	Diacritical mark wedge denotes the estimation
$L_{ij}$	Observer gain at the jth sampling time of the master (i=m)/slave (i=s) robot in task space
α,β…	Lowercase Greek letters indicate positive definite constant values
$e_{i}$	Position synchronization error of the master (i=m)/slave (i=s) robot in task space
$\Lambda_{i}$	Sliding mode switching parameters of the master (i=m)/slave (i=s) robot in task space
k	Kth joint in task space
$s_{i}^{}$	Sliding mode surface of the master (i=m)/slave (i=s) robot in task space
$s_{i}^{\Delta_{i}}$	Sliding surface switching band of the master (i=m)/slave (i=s) robot in task space
$\Delta_{i}$	Bandwidth of sliding mode surface switching band of the master (i=m)/slave (i=s) robot in task space
$\lambda_{i}$	Switching gain of sliding surface of the master (i=m)/slave (i=s) robot in task space
$\Phi_{i}$	Reaching law gain of sliding mode surface of the master (i=m)/slave (i=s) robot in task space
‖•‖	Euler norm
